# Novel Intravaginal Drug Delivery System Based on Molecularly PEGylated Lipid Matrices for Improved Antifungal Activity of Miconazole Nitrate

**DOI:** 10.1155/2018/3714329

**Published:** 2018-06-06

**Authors:** Franklin Chimaobi Kenechukwu, Anthony Amaechi Attama, Emmanuel Chinedum Ibezim, Petra Obioma Nnamani, Chukwuebuka Emmanuel Umeyor, Emmanuel Maduabuchi Uronnachi, Mumuni Audu Momoh, Paul Achile Akpa, Angela Chizoba Ozioko

**Affiliations:** ^1^Drug Delivery and Nanomedicines Research Group, Department of Pharmaceutics, University of Nigeria, Nsukka 410001, Enugu State, Nigeria; ^2^Nanomedicines and Drug Delivery Unit, Department of Pharmaceutics and Pharmaceutical Technology, Nnamdi Azikiwe University, Awka, Anambra State, Nigeria

## Abstract

The aim of this study was to investigate the potential of microparticles based on biocompatible phytolipids [Softisan® 154 (SF) (hydrogenated palm oil) and super-refined sunseed oil (SO)] and polyethylene glycol- (PEG-) 4000 to improve intravaginal delivery of miconazole nitrate (MN) for effective treatment of vulvovaginal candidiasis (VVC). Lipid matrices (LMs) consisting of rational blends of SF and SO with or without PEG-4000 were prepared by fusion and characterized and employed to formulate MN-loaded solid lipid microparticles (SLMs) by melt-homogenization. The SLMs were characterized for physicochemical properties, anticandidal activity, and stability. Spherical discrete microparticles with good physicochemical properties and mean diameters suitable for vaginal drug delivery were obtained. Formulations based on SO:SF (1:9) and containing highest concentrations of PEG-4000 (4 %w/w) and MN (3.0 %w/w) were stable and gave highest encapsulation efficiency (83.05–87.75%) and inhibition zone diameter (25.87±0.94–26.33±0.94 mm) and significantly (p<0.05) faster and more powerful fungicidal activity regarding killing rate constant values (7.10 x 10^−3^–1.09 x 10^−2^ min^−1^) than commercial topical solution of MN (Fungusol®) (8.00 x 10^−3^ min^−1^) and pure MN sample (5.160 x 10^−3^ min^−1^). This study has shown that MN-loaded SLMs based on molecularly PEGylated lipid matrices could provide a better option to deal with VVC.

## 1. Introduction

Over the past two decades, there has been a dramatic increase in the rate of vulvovaginal candidiasis (VVC), a common fungal infection caused by abnormal growth of yeast-like fungi (*Candida albicans*) on the female genital tract mucosa [1] and often characterized by painful and uncomfortable manifestations such as intense itching, irritation, vaginal discharge, erythema, and dysuria [2–4], with associated diminished quality of life of many women, considerable morbidity, and high and escalating healthcare cost to the society [5]. The total annual cost for dealing with VVC in USA alone was estimated to be at 1.8 billion US dollars [6]. In Africa, prevalence are higher in women treated with broad-spectrum antibiotics, pregnant women, diabetic women, and women with human immunodeficiency virus/acquired immunodeficiency syndrome (HIV/AIDS) [7, 8]. Approximately 75 % (three-quarters) of all women experience at least one episode of VVC during their lifetime and nearly half of them suffer from multiple episodes [9]. The infection most often reoccurs; hence patients receive therapy over a long time [1, 10]. It has been suggested that the cause of relapse in women with VVC is the reemergence of* C. albicans* from deeper layers of vaginal tissue [9, 11]. The high incidence, relapse, and associated healthcare cost of VVC highlight the need for the development of effective agents for its treatment.

In recent years, the most prescribed treatment for VVC has been local vaginal administration of imidazole antifungals, with associated advantages including the ability to deliver the active agent directly to the site of action, maintenance of the required concentration of active substance at the site of action for prolonged period, avoidance of numerous side effects of systemically applied drugs, and avoidance of extensive first pass metabolism [12–17]. Miconazole nitrate (MN), which is one of the imidazole antifungals widely and effectively used for the treatment of VVC, has a dual mechanism of action: the inhibition of ergosterol biosynthesis and the inhibition of peroxidases, which causes the accumulation of peroxide within the cell, leading to cell death [5, 18, 19]. Unfortunately, per oral use of MN is unacceptable due to severe side effects, including gastrointestinal disturbances. Thus, topical administration of MN is recommended. Nevertheless, the conventional MN topical vaginal delivery systems [vaginal solutions, cream, gels, and inserts (vaginal tablets and pessaries)] suffer from a lot of disadvantages including uncomfortable feeling to the user and side effects on the application site like burning, redness, and swelling [20, 21]. Furthermore, due to the immediate release of MN from these formulations they can stimulate the immune system of body generating high impact allergic reactions [22]. Deep-seated fungal infections such as recurrent VVC may be more difficult to treat because the drug released from conventional topical formulation cannot reach the target site due to the low penetration capacity. In addition, being a biopharmaceutical classification system (BCS) class II drug, MN is limited by its very poor aqueous solubility (< 1 *μ*g/mL) as well as drug resistance [19, 23], which poses some difficulties in its dosage design and leads to therapeutic failure. The entrapment of MN in suitable vehicle would assist in its localized delivery and an improved solubility and availability at the targeted site coupled with a reduction in its associated undesirable side effects, which would culminate in administration of low doses and amelioration of the possibility of resistance.

Previous approaches which have been used to overcome the limitation of poor water solubility and low bioavailability of MN include the addition of surfactants, coprecipitation techniques, or incorporating MN into various novel carrier systems [23–30]. PEGylation is one of the most promising and extensively studied strategies for improving the performance of drugs and bioactives and one of its characteristics is the highly targeted use of very small quantities of substance for therapy, especially of intracellular infections [31] including VVC; this offers huge potential for sustainable medicine. Reduction in dose is also envisaged in addition to dosage regimen modification [30]. PEGylation techniques have been used to enhance the therapeutic efficacy of drugs by enabling increased drug concentration and longer dwelling time at the site of action [32, 33]. In view of this, PEGylation could be employed to develop vaginal delivery system for sustained and controlled vaginal delivery of MN for effective treatment of VVC. Lipid-based medicines are highly tolerated (nontoxic) and can easily be manufactured industrially [34, 35]. Furthermore, PEG-lipid carriers have shown promise for enhanced drug delivery [36]. PEGylated lipids can be applied to drug formulations to prolong the half-life of the drugs [37]. An interesting alternative approach for MN encapsulation, therefore, would be the use of PEGylated lipid-based carriers since these hydrolipophilic carriers would ensure close contact to deeper layer of the vaginal mucosa and would increase the amount of MN deposited on the vaginal skin and penetration into the vaginal skin layers as well as enhance duration of MN action for effective treatment of VVC. Hence vaginal delivery of MN using these PEGylated lipid-based carriers would provide high local tissue levels, more rapid drug delivery, lower systemic exposure, and prolonged localized activity. This may be especially important for treating pregnant patients suffering from VVC. Previous study by our research group demonstrated that PEGylated solid lipid microdispersions of gentamicin (an aminoglycoside antibiotic) were quite useful for skin targeting via topical route and notably offered localized antibacterial effect [38]. Consequently, exploring the potential of tailor-made drug delivery carriers such as lipid-based PEGylated delivery system in improving the topical vaginal delivery of antifungal drugs such as MN for localized treatment of VVC seems worthwhile.

At the moment, there is paucity of information in the literature on the use of PEGylated solid lipid microparticles for enhancement of the intravaginal delivery and antimycotic activity of MN against* C. albicans*. The exploration of PEGylated structured phytolipids [Softisan® 154 (a hydrogenated vegetable oil) and super-refined sunseed oil fortified with vitamin A] in the development of an effective intravaginal delivery system for MN, in order to maximize efficacy, reduce the drug's adverse effects, and reduce incidence of relapse following VVC therapy, informs the aim of this study. Therefore, the purpose of this study was to formulate and characterize intravaginal PEGylated solid lipid microparticles for enhanced antimycotic activity of MN against* C. albicans*.

## 2. Materials and Methods

### 2.1. Materials and Reagents

The pure sample of miconazole nitrate used was purchased from Gutic Biosciences Limited, India. Soluplus® (polyvinylcaprolactam-polyvinyl acetate-polyethylene glycol grafted copolymer) was kindly provided by BASF (Ludwigshafen, Germany). Other materials include methanol and ethanol (Sigma Aldrich, USA), sorbic acid (Foodchem Int. Co., China), Polysorbate® 80 (Tween® 80) (Merck KGaA, Darmstadt, Germany), Sabouraud Dextrose Agar (SDA) (United Technology Trade Corp, USA), polyethylene glycol 4000 (PEG 4000) (Ph. Eur. Carl Roth GmbH + Co. KG Karlsruhe, Germany), Softisan® 154 (Cremer Oleo GmbH, Hamburg, Germany), sunseed oil (double refined) vitamin A fortified (Kelwaram Chanrai Group, Lagos, Nigeria), Fenicol® 250 mg (chloramphenicol, Sam Pharm. Ltd., Ilorin, Nigeria), and distilled water (Lion water, Nigeria). The brand of commercially available miconazole nitrate lotion used was Fungusol® lotion (marketed by AfrabChem Ltd., Lagos, Nigeria).* C. albicans* was isolated from high vaginal swab (HVS) of an immunosuppressed female HIV-positive patient of Bishop Shanahan Hospital, Nsukka, Nigeria. Informed patient's consent was sought prior to collection. All other chemicals and reagents used were of analytical grade and obtained commercially.

### 2.2. Preparation of Lipid Matrices

Lipid matrices were prepared by fusion in paraffin oil bath using a hot plate (IKA RCT basic, IKA, Staufen, Germany), employing super-refined sunseed oil and Softisan® 154 at various ratios (SO:SF ratios 1:1, 1:3, 1:6, and 1:9). In each case, appropriate quantities of the lipids were weighed, melted together in a temperature-regulated oil bath at 70°C, and stirred until a homogenous melt of each admixture was obtained, which was then stirred at room temperature until solidification. After 24 h, various quantities (90, 80, and 60 %w/w) of each prepared lipid matrix were melted together with corresponding amounts of polyethylene glycol (PEG 4000) (10, 20, and 40 %w/w) incorporated at 80°C over the oil bath to give PEGylated lipid matrices containing 1:9, 2:8, and 4:6 ratios of PEG:lipid matrix, respectively (each for 1:1, 1:3, 1:6, and 1:9 base matrices), which were stirred properly and allowed to solidify also. Thus sixteen lipid matrices comprised of four non-PEGylated lipid matrices [i.e., LM 1:1, LM 1:3, LM 1:6, and LM 1:9] and twelve PEGylated lipid matrices were formulated as shown in Table 1. The lipid matrices were thereafter stored in airtight and moisture resistant glass bottles away from light until used.

### 2.3. Thermal Analysis of Lipid Matrices

Melting transitions and changes in heat capacity of all the lipid matrices were determined using a differential scanning calorimeter (DSC Q100 TA Instrument, Germany). About 5 mg of each lipid matrix was weighed into an aluminum pan, hermetically sealed, and the thermal behaviour determined in the range of 20–250°C at a heating rate of 5°C/min. The temperature was held at 80°C for 10 min and thereafter cooled at the rate of 5 to 10°C/min. Baselines were determined using an empty pan, and all the thermograms were baseline-corrected. Lipid matrices with desirable thermal properties were selected and further analyzed.

### 2.4. Drug-Lipid Matrix Compatibility Study

Drug-lipid matrix compatibility study performed by polarized light microscopy (PLM) was done to screen the drug and the excipients for solubility determination [39]. Each selected lipid matrix was melted by fusion in paraffin oil bath at 80°C using a thermoregulated hot plate, after weighing appropriate amount and different concentrations (0.5, 1.0, 2.0, 3.0, and 5.0 %w/w) of MN in 10.0 %w/w of each selected lipid matrix were weighed in an analytical balance and added to the melted lipid, followed by stirring using a magnetic stirrer-hot plate assembly at 100 rpm for 10 min. The drug-lipid matrix mixtures were further observed thereafter using a microscopic slide (Marinfield, Germany) and polarized light microscope (Lieca, Germany) attached with a Motic image analyzer (Moticam, China). Photomicrographs of samples were thereafter taken at x 1000 magnification.

### 2.5. Preparation and Characterization of Drug-Loaded Lipid Matrices

Drug-loaded lipid matrices were prepared by fusion, based on the result of drug-lipid matrix compatibility screening performed earlier by PLM, which indicated solubility limit of 3 %w/w of MN in the lipid matrix. With target lipid matrix concentration of 10 %w/w in the SLMs to be developed, 1.0 g of each optimized lipid matrix and 0.3 g of MN were used. The obtained drug-loaded PEGylated and non-PEGylated lipid matrices (representative batches) were stored until analyzed by Fourier transform infrared (FT-IR) spectroscopy. Briefly, FT-IR spectroscopic analysis was conducted using a Shimadzu FT-IR 8300 Spectrophotometer (Shimadzu, Tokyo, Japan) and the spectrum was recorded in the wavelength region of 4000 to 400 cm^−1^ with threshold of 1.303, sensitivity of 50, and resolution of 2 cm^−1^ range. A smart attenuated total reflection (SATR) accessory was used for data collection. The potassium bromate (KBr) plate used for the study was cleaned with a trisolvent (acetone-toluene-methanol at 3:1:1 ratio) mixture for baseline scanning. A 0.1 g of each sample was mixed with 0.1 ml nujul diluent. The solution was introduced into the potassium bromate (KBr) plate and compressed into discs by applying a pressure of 5 tons for 5 min in a hydraulic press. The pellet was placed in the light path and the spectrum obtained. Spectra were collected in 60 s using Gram A1 spectroscopy software, and the chemometrics were performed using TQ Analyzer1. The study was also carried out using pure sample of MN.

### 2.6. Optimization of Formulation Parameters

Various batches of the unloaded SLMs were formulated by melt-homogenization method [40] by varying the stirring speed and time. Four lipid matrices were optimized based on detailed characterization earlier carried out, namely, one non-PEGylated lipid matrix (LM 1:9) and three PEGylated lipid matrices [PEG-LM 1:9 (1:9), PEG-LM 1:9 (2:8), and PEG-LM 1:9 (4:6)]. These optimized lipid matrices were used to prepare SLMs containing MN after optimization of the formulation parameters (stirring speeds and times: 5,000 rpm for 10 min, 10,000 rpm for 5 min, and 10,000 rpm for 7.5 min). Briefly, each optimized lipid matrix (10 g) was melted over a liquid paraffin oil bath in a temperature-regulated hot plate and maintained at a temperature of 80°C. Soluplus®, sorbic acid, and Tween® 80 were dispersed in distilled water and maintained at the same temperature as the lipid matrix to form the aqueous phase. The two phases were mixed and homogenized using the Ultra-Turrax homogenizer (T 25 digital Ultra-Turrax; IKA, Staufen, Germany) at various homogenization speeds and times. The formulations were analyzed by PLM from which 5,000 rpm for 10 min was chosen for the preparation of the drug-loaded SLMs.

### 2.7. Formulation of SLMs

PEGylated and non-PEGylated SLMs of MN were prepared using the optimized drug concentrations (1.0, 2.0, and 3.0 %w/w for MN), optimized stirring speed and time (5,000 rpm for 10 min), optimized lipid matrices [LM (1:9), PEG-LM 1:9 (1:9), PEG-LM 1:9 (2:8), and PEG-LM 1:9 (4:6)], Tween® 80 (surfactant), sorbic acid (preservative), Soluplus® (solubilizer/cosurfactant), and distilled water by the melt-emulsification technique (Kenechukwu et al. 2016). The formulation followed the procedure described previously but with the addition of the drug in the molten lipid matrix before mixing with aqueous phase and subsequent homogenization. Unloaded (zero-drug) PEGylated and non-PEGylated SLMs were also prepared to serve as controls. The formulation compositions of the various batches of MN-loaded SLMs are shown in Table 2.

### 2.8. Characterization of SLMs

#### 2.8.1. Determination of Percentage Yield

The practical yields of the SLMs dispersions from each lipid matrix were determined to evaluate the efficiency of the method of preparation by measuring their weights. The percentage (%) yield was calculated using [38]

(1) $$\begin{array}{c} Percentage\;\;recovery=\frac{W_{1}}{{W_{2}+W}_{3}}\;X\;100 \end{array}$$

where W_1_ is the weight of the SLMs formulated (g), W_2_ is the weight of the drug added (g), and W_3_ is the weight of the lipid and other excipients (g).

#### 2.8.2. Determination of Encapsulation Efficiency and Loading Capacity

Approximately 5 ml of the SLMs was added to a microconcentrator (Vivaspin® 6, Vivascience, Hanover, Germany) consisting of filter membrane with molecular weight cut-off (MWCO) of 10,000 Daltons, centrifuged (TDL-4 B. Bran Scientific and Instru. Co., London, England) at 3000 rpm for 3 h and the supernatant was collected and properly diluted with appropriate solvent. The dilute solution was analyzed for drug content spectrophotometrically (Unico 2102 PC UV/Vis Spectrophotometer, New York, USA) at predetermined wavelength of 285 nm. The amount of drug encapsulated in the SLMs was calculated with reference to standard Beer-Lambert's plot for MN to obtain the EE % using [41]

(2) $$\begin{array}{c} EE\;\left(\;\%\;\right)=\frac{Actual\;\;drug\;\;content}{Theoretical\;\;drug\;\;content}\times 100 \end{array}$$

LC expresses the ratio between the entrapped active pharmaceutical ingredient (API) and total weight of the lipids [32]. It was determined using

(3) $$\begin{array}{c} LC=\frac{W_{a}}{W_{1}}\;X\;100 \end{array}$$

where W_l_ is the weight of lipid added in the formulation and W_a_ is the amount of API entrapped by the lipid.

#### 2.8.3. Determination of pH

The pH of each of the SLM dispersions was measured using a validated digital pH meter (pH ep Hanna Instruments, Padova, Italy) after calibrating with standard buffers. The electrode component was immersed into 50 ml quantities of the dispersions and the reading recorded. Each measurement was performed in triplicate and the average calculated.

#### 2.8.4. Continuous Shear Viscometry

The viscosity of each optimized SLMs formulation was measured using a digital viscometer (NDJ-5S Viscometer, Labsciences, England), with spindle number 02 (for less viscous fluids) at different speeds (6, 12, 30, and 60 rpm). The spindle was immersed in the sample in a 20-mL beaker and attached to the coupling nut such that the SLMs level was at the groove on the shaft (spindle guard leg was not used). The test was run at ambient laboratory temperature (32°C) and the viscosity (mPa.s) and percent torque at each speed of rotation were recorded.

#### 2.8.5. Determination of Morphology and Surface Characteristics

The particle size and morphology of the SLMs were determined by PLM. Briefly, approximately a drop of the SLMs from each batch was placed on a slide (Marinfield, Germany) using a 1 ml dropper. It was then covered with a cover slip and viewed under a polarized light microscope (Lieca, Germany) attached with a Motic image analyzer (Moticam, China). With the aid of the software in the microscope, the particle morphologies were observed and photomicrographs were taken at x 1000 magnification. The sizes of the particles were measured and the average taken.

#### 2.8.6. FT-IR Spectroscopy

FT-IR spectroscopic analysis was conducted on the SLMs to evaluate possible molecular interactions between the drug and the excipients. The potassium bromate (KBr) plate used for the study was cleaned with a trisolvent (acetone-toluene-methanol at 3:1:1 ratio) mixture for baseline scanning. A 0.1 ml volume of each batch of the SLMs was mixed with 0.1 ml nujul diluent. The solution was introduced into the potassium bromate (KBr) plate and compressed into pellets, each of which was placed in the light path and the spectrum obtained. All other aspects of the protocols involved in FT-IR spectroscopy were the same as those outlined above.

#### 2.8.7. Preliminary Anticandidal Susceptibility Study

The modified Sabouraud dextrose agar (SDA) used as medium for the study was prepared using the manufacturer's standard with slight modification. The* C. albicans* for the study was isolated from high vaginal swab (HVS) of an immunosuppressed female HIV-positive patient of Bishop Shanahan Hospital, Nsukka, Nigeria, using sterile swab stick and sterile vaginal speculum. Informed patient's consent was sought. The specimen collected was analyzed using standard World Health Organization (WHO) laboratory procedures [42], purified and kept on SDA agar slant as bench culture at 4°C. The overnight culture of* C. albicans *was suspended in sterile water, and the turbidity was compared with 0.5 MacFarland turbidity standards and used for the test.

For the determination of inhibition zone diameter (IZD) of the SLMs, the* C. albicans* was suspended in SDA using sterile water and incubated at 25°C for 24 h. The plate agar well diffusion method was used for the* in vitro* anticandidal evaluation. This method depends on the diffusion of antibiotics from holes on the surface of the microbial seeded agar [43]. Precooled sterile molten SDA (20 ml) was inoculated with standardized suspension (0.1 ml) of* C. albicans* broth culture, mixed thoroughly, poured into sterile Petri dishes, and rotated for even distribution of the organism. The agar plates were allowed to set and a sterile cork borer (7 mm diameter) was used to bore three cups in the seeded agar medium. A 0.1 ml volume (100 *μ*L) each of the samples was added respectively into the different cups in each of the plates using a micropipette with disposable tips. The plates were allowed to stand at room temperature for 15 min to enable the samples diffuse into the medium before incubating at 25°C for 48 h. The diameter of each inhibition zone was measured and the average determined. The SLMs with highest IZDs were selected for further studies.

#### 2.8.8. Determination of Minimum Inhibitory Concentrations (MICs) and Minimum Fungicidal Concentrations (MFCs) of the Optimized SLMs

These were carried out by the agar dilution technique [44, 45]. The optimized drug-loaded SLM formulations were individually twofold serially diluted with sterile water to obtain several (12) dilutions [that ranged from 15,000 to 1.831056 *μ*g/ml for optimized MN-loaded SLMs using 3 %w/v (i.e., 30 mg/ml stock solution)]. Similarly, stock solution of pure MN sample (30 mg/ml of MN) was prepared using dimethyl sulfoxide (DMSO) and sterile water and twofold serially diluted, together with Fungusol® lotion. Then 0.1 mL of* C. albicans* suspension (10^7^ cfu/ml) was individually added to 1.0 ml of each of the solution after addition of 1.0 ml double strength of SDA fortified with 0.03 % chloramphenicol (250 mg/10 ml ethanol). The mixture was poured into a sterile Petri dish, rocked, allowed for 20 min for prediffusion, and incubated, after which the Petri dishes were examined for microbial growth. The highest dilution (i.e., lowest concentration) at which no fungal growth was detected on the surface of the solidified agar was recognized as the MIC. After completing the MIC determination, the agar plates showing absence of growth in the MIC test were identified and used for MFC test. Here, discs were cut from each agar plate and transferred into corresponding containers of the fresh nutrient medium and thereafter incubated for 48 h. At the end of incubation, the highest dilution at which there was no fungal growth on the SDA plates was identified as the MFC.

#### 2.8.9. Killing Rate Study on the Optimized Drug-Loaded SLMs

A modification of the killing rate method described elsewhere [46] was used. In therapy, as a rule of thumb, clinicians attempt to have peak* in vivo* antimicrobial levels in tissue that exceed the minimal microbicidal concentration; hence a concentration in each case that is four times the MFC as determined in the preceding section was arbitrarily selected for each sample to examine the kinetics of killing by the optimized SLMs. A 0.1 ml volume of standardized culture of* C. albicans *was mixed with 9.9 ml of each sample of specified concentration (4 x MFC) and that formed the reaction mixture, which was stored in a thermoregulated laboratory condition. Plain SLM formulation was used as a negative control. At regular time intervals (15, 30, 45, 60, 75, 90, 105, and 120 min), 1 ml replicate samples were collected from the reaction mixture for viable cell counting. The action of the drug in the collected sample was stopped immediately on withdrawal by the addition of 50 *μ*L of sodium thiosulphate (inactivating/quenching agent) and then made up to 10 ml (first tenfold dilution). From this dilution, three tenfold serial dilutions were also obtained, giving a total of four tenfold serial dilutions of the collected samples (i.e., 10^1^, 10^2^, 10^3^, and 10^4^). A drop of each dilution was added on the modified SDA plate in replicates and incubated at 25°C for 48 h. The colonies of* C. albicans *were counted and used to determine the colony forming unit per milliliter (cfu/ml) employing

(4) Colony  forming  unit  per  ml=Mean  colony  count  per  drop X RDFVolume  of  one  drop

(5) CFU/ml=Mean  colony  count  per  drop X RDF0.015  ml

where RDF means reciprocal of dilution factor.

The killing rate constants (K) of the optimized SLMs were calculated from the slope of the semilogarithmic plot of the population of microbial cells (survivors) at various time t of each batch against time using linear regression analysis employing (6) (Okore 2005). D-values (time required to reduce the microbial population by 90 %) of the optimized SLMs were also obtained using equation (7) [47].

(6) log⁡Nt=log⁡N0–Kt

(7) D−value=tlog⁡N0−log⁡Nt

where N_0_ is the population of microbial cells at zero time; N_t_ is the population of survivors at time, t; K is the killing rate constant; and D is the D-value.

#### 2.8.10. Stability Studies on the SLMs

The pH and particle size of the SLMs from each batch were determined in a time-dependent manner after one week, one month, and three months of storage at room temperature [38].

### 2.9. Data and Statistical Analysis

All experiments were performed in replicates for validity of statistical analysis. Results were expressed as mean ± SD. Student's t-test was performed on the data sets generated using Statistical Package for Social Sciences (SPSS) software, version 12 (Chicago, IL). Differences were considered significant at p < 0.05.

### 2.10. Compliance with Ethical Standards

*C. albicans* was isolated from high vaginal swab (HVS) of an immunosuppressed female HIV-positive patient of Bishop Shanahan Hospital, Nsukka, Nigeria. Informed patient's consent was sought prior to collection. All institutional and national guidelines for the collection of specimens from human subjects were followed.

## 3. Results and Discussion

### 3.1. Thermal Characterization

**Figure 1:**
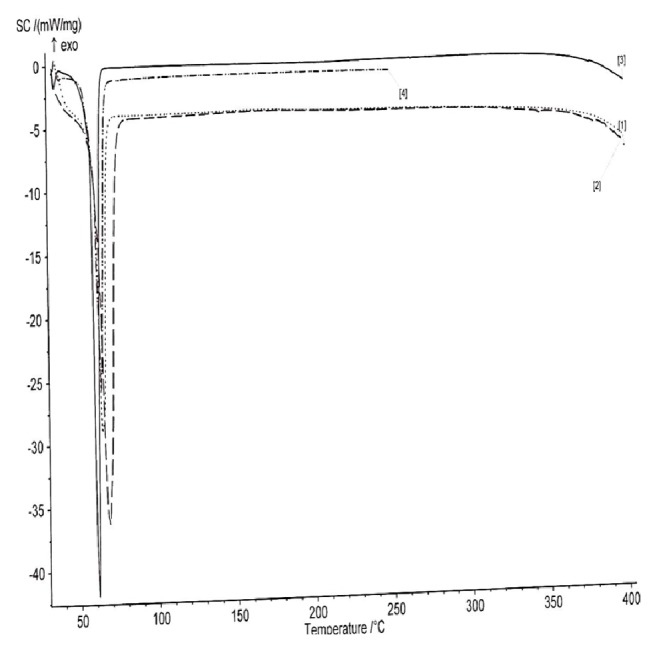
DSC thermograms of Softisan® 154, PEG 4000, optimized PEGylated and non-PEGylated lipid matrices (representative batch) in superposition. Key: 1 = PEG-LM, 2 = LM, 3 = Softisan® 154, and 4 = PEG 4000, and LM means lipid matrix prepared with super-refined sunseed oil and Softisan® 154, while PEG-LM means PEGylated lipid matrix made with LM and PEG 4000.

**Figure 2:**
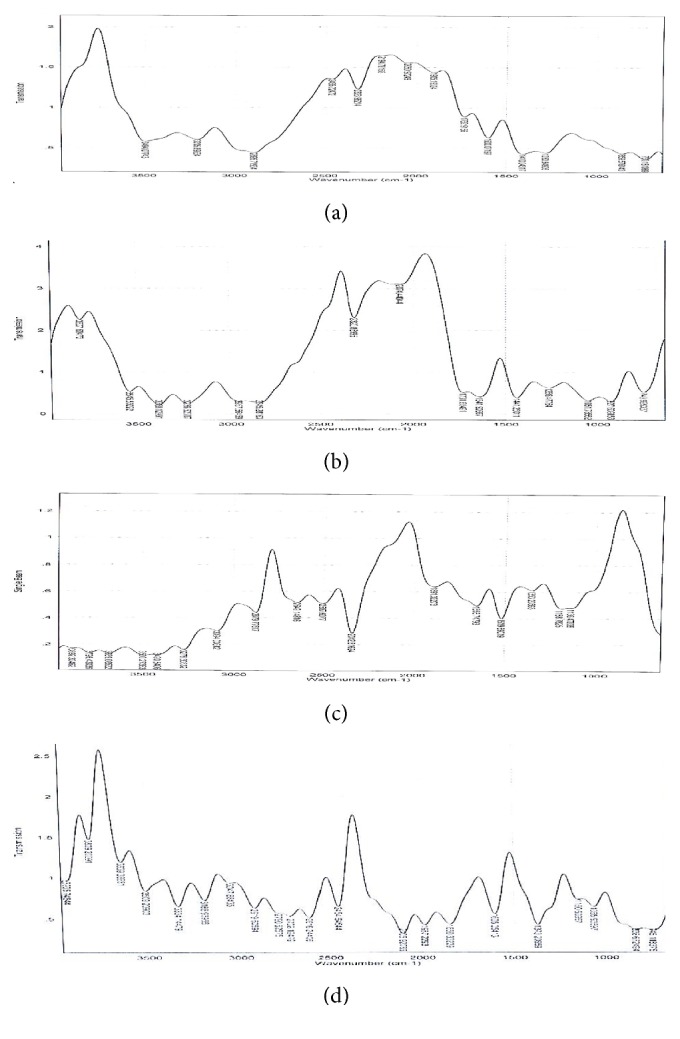
Fourier transform infrared (FT-IR) spectra of miconazole nitrate (MN) (a) and MN-loaded non-PEGylated and PEGylated lipid matrices (b and c) and selected miconazole nitrate-loaded SLMs (d). Note: non-PEGylated lipid matrix (LM 1:9) prepared with super-refined sunseed oil (10 %w/w) and Softisan® 154 (90 %w/w) and PEGylated lipid matrix [PEG-LM 1:9 (4:6)] made with PEG 4000 (40 %w/w) and LM 1:9 (60 %w/w) were used.

Figure 1 shows the DSC thermograms of Softisan 154, PEG-4000, and optimized lipid matrices (PEGylated and non-PEGylated) in superposition while Table 3 shows the thermal properties of all the matrices. The DSC thermogram of Softisan® 154 showed a sharp melting peak of 61.4°C with an enthalpy of -8.9 mW/mg, indicating its crystalline nature. Admixture of Softisan® 154 (SF) and sunseed oil (SO) at equal ratio (1:1) led to a decrease in the melting point (60.82°C) but an increase in enthalpy (-9.0 mW/mg) compared with Softisan® 154. An increase in the ratio of Softisan® 154 in the lipidic blends (1:3, 1:6, and 1:9 of SO:SF) gave rise to increase in melting points [61.62°C (LM 1:3), 115.18°C (LM 1:6), and 64.56°C (LM 1:9)] with corresponding decrease in enthalpies [-2.5 mW/mg (LM 1:3), -0.25 mW/mg (LM 1:6), and -2.05 mW/mg (LM 1:9)]. The (DSC) melting endothermic peak of PEG 4000 was 64.40°C with an enthalpy of -36.36 mW/mg indicating its crystalline nature, while DSC thermograms of PEGylated LMs showed different melting peaks and thermal properties. PEGylated lipid matrix based on 1:1 ratio of SF and SO gave melting points of 142.49°C [PEG-LM 1:1 (1:9)], 145.66°C [PEG-LM 1:1 (2:8)], and 62.66°C [PEG-LM 1:1(4:6)] with corresponding enthalpies of -0.35 mW/mg [PEG-LM 1:1 (1:9)], -0.4 mW/mg [PEG-LM 1:1 (2:8)], and -2.0 mW/mg [PEG-LM 1:1 (4:6)]. The DSC result of PEGylated lipid matrices based on 1:3 ratio of SO and SF showed melting peaks of 88.0°C [PEG-LM 1:3 (1:9)], 60.60°C [PEG-LM 1:3 (2:8)], and 65.76°C [PEG-LM 1:3 (4:6)] with corresponding enthalpies of -0.35 mW/mg [PEG-LM 1:3 (1:9)], -2.3 mW/mg [PEG-LM 1:3 (4:6)], and -3.4 mW/mg [PEG-LM 1:3 (2:8)]. However, the results showed that all PEGylated adducts of LM (1:6) had lower melting peaks {61.51°C [PEG-LM 1:6 (1:9)], 61.57°C [PEG-LM 1:6 (2:8)], and 61.75°C [PEG-LM 1:6 (4:6)]} with corresponding higher enthalpies {-4.0 mW/mg [PEG-LM 1:6 (1:9)], -3.25 mW/mg [PEG-LM 1:6 (2:8)], and -4.5 mW/mg [PEG-LM 1:6(4:6)]} than non-PEGylated LM 1:6. More so, the DSC thermograms of all PEGylated adducts of LM 1:9 generally lowered the melting points {60.75°C [PEG-LM 1:9 (1:9)], 61.32°C [PEG-LM 1:9 (2:8)], and 61.12°C [PEG-LM 1:9 (4:6)]} better than other lipid matrices, even though they had similar enthalpies {-4.25 mW/mg [PEG-LM 1:9 (1:9)], -3.6 mW/mg [PEG-LM 1:9 (2:8)], and -4.1 mW/mg [PEG-LM 1:9 (4:6)]}. Further details of the thermal properties could be found in supplementary files 1 and 2 (Figs.  1 and 2).

DSC is an important technique that provides necessary information concerning the thermal behaviour and crystalline structure of a material. Thermal behaviour can provide information about the thermal stability, phase separation, and interactions in polymeric and lipid systems. This valuable information is necessary to establish the possibility of modifying the properties of polymers and lipids and also to confirm the stability of drugs in polymeric or lipidic systems [23, 36, 38]. Since higher melting point or enthalpy values indicate more ordered crystal structures [38], it follows that the interaction between the fatty acid contents of SO (which is a super-refined unsaturated oil from sunflower) and Softisan® 154 (super-saturated hydrogenated palm oil) resulted in the partly disordered crystalline arrangement which suggests great deformation in the lattice structure [38]. Higher amount of the liquid lipid (SO) in the LM gave rise to a relatively amorphous structure as shown by the lower melting peak of LM (1:1) compared to other LMs. On the other hand, increased amount of the solid lipid led to decreased amorphicity of the LMs 1:3, 1:6, and 1:9; they gave lower enthalpies than both SF and LM 1:1. By implication, all the lipid matrices generated had an imperfect matrix structure (due to distortion of crystal arrangement of the bulk lipid after melting and solidification), which would have created numerous spaces for drug localization.

Upon addition of PEG 4000 into the lipid matrices, there was further modification in the crystal properties of the lipid matrices. Moreover, increased amount of PEG 4000 initially led to an increase in melting point but further increase led to a drastic (sharp) decrease in melting point. Results showed that there were both increase and decrease in the melting points as well as the enthalpies compared with non-PEGylated adduct (LM 1:3). However, all PEGylated adducts of LM (1:6) had lower melting peaks with corresponding higher enthalpies than non-PEGylated LM 1:6 Also, increased amount of PEG 4000 led to a slight increase in the melting point, while the enthalpies initially decreased and then increased slightly with increased PEG 4000 content. PEG-LM (1:9) showed the greatest enhancement in the disorderliness of the lipid matrices, which would result to increased drug incorporation and holding capacity of the LM (1:9), consistent with previous reports [38, 48]. In addition, the DSC thermograms of LM (1:9) and its PEGylated adducts are the most consistent in terms of the melting temperature range; they melted over the greatest (widest) temperature range (25–75°C).

**Figure 3:**
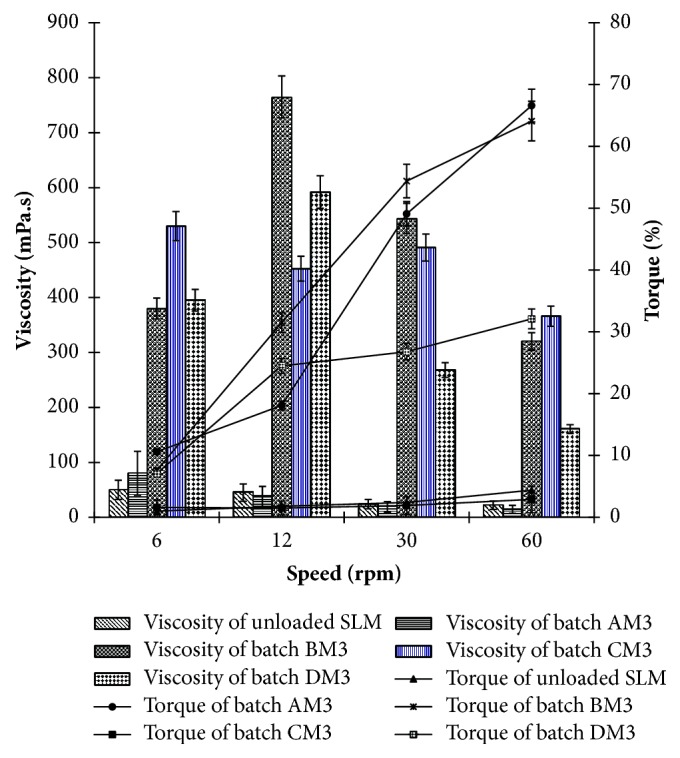
Viscosity profiles of optimized miconazole nitrate-loaded SLMs. Key: batch AM_3_ is optimized MN-loaded non-PEGylated SLMs containing 3.0 %w/w of miconazole nitrate, and batches B-D are PEGylated SLMs containing increasing concentrations (1.0, 2.0 and 4.0 %w/w) of PEG 4000, respectively; AM_3_–DM_3_ contain 3.0 %w/w of miconazole nitrate, while unloaded is plain SLM.

Result of drug-lipid compatibility (static crystallization of the lipid-drug admixture) studied by PLM (not shown) indicate that the solubility limit of miconazole nitrate in the lipid matrices (PEGylated and non-PEGylated) was 3.0 %w/w while the drug crystallized out of the matrices at 5.0 % MN). Result of drug-lipid compatibility revealed that MN and the lipid matrices were compatible. During crystallization, the material goes through different stages beginning with nucleation and followed by unhindered growth and structure formation. The initial nucleation is characterized by the appearance of nuclei far apart from each other leading to nonuniform crystallization, which may result in mixture of crystals and low crystal order capable of increasing drug loading capacity of the material [39]. The solubility limit of miconazole nitrate in the lipid matrices (PEGylated and non-PEGylated) was determined to be 3.0 %w/w. The rate of crystallization of the drug would affect the ultimate size of the microparticle. If the drug crystallizes faster than the lipid matrix (as was the case with 5.0 % MN), encapsulation efficiency is expected to be higher and* vice versa* [39]. Further details of the results of the static crystallization could be found in the supplementary file 3 (Fig.  3).

### 3.2. FT-IR Spectroscopic Analysis of MN-Loaded Lipid Matrices

The FT-IR spectra of MN-loaded lipid matrices are presented in Figures 2(a)–2(c). It could be seen from the figure that the FT-IR spectrum of pure MN sample showed principal characteristic absorption bands at 2494 cm^−1^ (N-H stretching of benzene ring), 3205 cm^−1^ (OH group), 2895 cm^−1^ (C-H stretching of benzene ring), 2323 cm^−1^ (C=N stretching), 1732 cm^−1^ (carbonyl compound: CHO), 1600 cm^−1^ (conjugated *α*,*β*-unsaturated C=C bond in benzene ring), 1410 cm^−1^ (NO_2_: nitro group), 1283 (C-O ester in benzene ring), and 859 cm^−1^ and 730 cm^−1^ (aromatic C-Cl deformation). FT-IR of MN-loaded non-PEGylated lipid matrix showed principal characteristic absorption bands of MN at 3807 cm^−1^ and 3545 cm^−1^ (N-H stretching of benzene ring), 3388 cm^−1^ and 3236 cm^−1^ (OH group), 2845 cm^−1^ (C-H stretching of benzene ring), 2320 cm^−1^ (C=N stretching), 1641 cm^−1^ (conjugated *α*,*β*-unsaturated C=C bond in benzene ring), 1441 cm^−1^ (NO_2_: nitro group), 1266 cm^−1^ and 1051 cm^−1^(C-O ester in benzene ring), and 927 cm^−1^ and 749 cm^−1^ (aromatic C-Cl deformation). FT-IR spectrum of MN-loaded PEGylated lipid matrix showed principal characteristic absorption bands of MN at 3276 cm^−1^ (N-H stretching ), 3094 cm^−1^ (OH group), 2649 cm^−1^, 2507 cm^−1^ and 2879 cm^−1^ (C-H stretching), 2342 cm^−1^ (C=N stretching), 1656 cm^−1^ (conjugated *α*,*β*-unsaturated C=C bond in benzene ring), 1519 cm^−1^ (NO_2_: nitro group), and 1188 cm^−1^ and 1136 cm^−1^ (C-O ester in benzene ring).

The FT-IR spectroscopic analysis was carried out to evaluate the possible molecular interactions between the drug and the matrices in the solid state [23]. In order words, it was employed to rule out any strong interaction between MN and the lipid matrices used in the formulation of the SLMs, since the IR spectrum of any given compound is always unique and characteristic. FT-IR spectrum of MN-loaded non-PEGylated and PEGylated lipid matrices showed similar characteristic peaks of pure MN, indicating that there was no strong chemical drug-lipid or drug-polymer interaction [23]. The FT-IR spectra of MN-loaded lipid matrices revealed almost all the major bands in MN without affecting the characteristic peak positions and trends significantly, which indicates absence of well-defined adverse interactions between the drug and the lipids and PEG 4000 or formation of a new compound. Presence of other peaks in the FT-IR spectra of MN-loaded lipid matrices indicates the presence of the polymer (PEG 4000) and the lipids (Softisan® 154 and super-refined sunseed oil). Overall FT-IR results suggested absence of any incompatibility between MN and excipients used in the formulation [23].

### 3.3. Optimization of Parameters for SLMs Preparation

**Figure 4:**
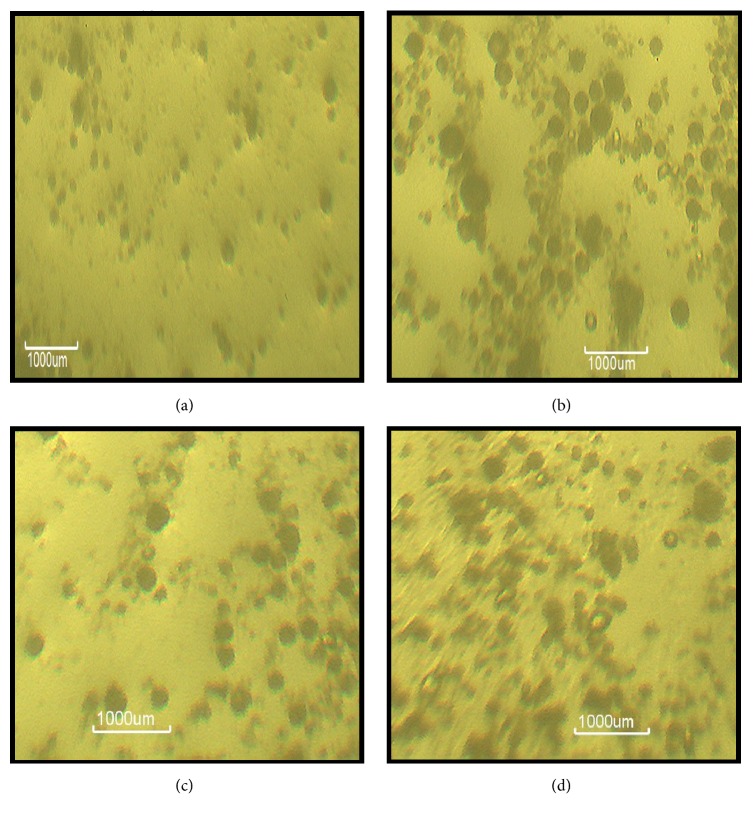
Polarized light micrographs of representative plain SLM (C_0_) (a) and non-PEGylated MN-loaded SLM (AM_1_) (b) and PEGylated MN-loaded SLMs (BM_3_ and DM_1_) (c and d) dispersions.

Polarized light micrographs of SLMs formulated at 5,000 rpm for 10 min, 10,000 rpm for 5 min, and 10,000 rpm for 7.5 min (not shown) indicated that at 10,000 rpm for 5 min, there was formation of bridges and caking. Formation of flocculated particles resulted. At 10,000 rpm for 7.5 min, there was shear-induced coalescence. However, more discrete and spherical particles were formed at 5,000 rpm for 10 min. Further details of the results could be found in the supplementary file 4 (Fig.  4).

PEGylated and non-PEGylated SLMs of MN were prepared using the optimized drug concentrations (1.0, 2.0, and 3.0 %w/w) (based on PLM and FT-IR analyses), formulation parameters (5000 rpm for 10 min) (based on PLM analysis), and lipid matrices [LM (1:9), PEG-LM 1:9 (1:9), PEG-LM 1:9 (2:9), and PEG-LM 1:9 (4:6)] (based on DSC analysis). Soluplus® was added as a cosurfactant and solubilizer, sorbic acid as a preservative, and Tween® 80 as a surfactant. The stirring speed and time (5,000 rpm for 10 min) were optimized and chosen for the preparation of the API-free and drug-loaded SLMs.

Homogenization speed and time were varied in order to optimize the formulation parameters for the SLMs. Physical stability of SLMs is affected by particle size [40] and size distribution is affected by stirring rate, temperature, and type and amount of polymers and/or lipids as well as viscosity of the continuous phase [48]. Formation of flocculated particles resulted at 10,000 rpm for 5 min and the SLMs formed at this homogenization speed and time might be good for the preparation of creams. At 10,000 rpm for 7.5 min, there was shear-induced coalescence which could be explained as follows: as dead ends of the lipid particles (small particles) are exposed to same or constant concentration of surfactant, with increased shearing time, shear-induced coalescence might result giving rise to larger particle sizes observed. However, more discrete and spherical particles were formed at 5,000 rpm for 10 min, and these SLMs would be good for the preparation of gels, as the SLMs would disperse more easily in the hydrogels. Particle micrograph of the optimized formulation parameters (5,000 rpm for 10 min) showed structured spherical and more discrete smaller but uniformly distributed particle sizes spread all over the formulation. Moreover, absence of particle aggregation in the formulation is indicative of physical stability of the optimized formulation based on the homogenization parameter [38].

### 3.4. Percentage Recovery, Encapsulation Efficiency, and Loading Capacity

The percentage of the SLMs recovered from the formulations (as presented in Table 4) indicates that both MN-loaded and unloaded SLMs had high percentage yields (94.76–99.10 %). There was insignificant difference (p > 0.05) between the percentage recovery of MN-loaded and unloaded SLMs as well as between PEGylated and non-PEGylated SLMs. The results of the encapsulation efficiency (EE%) and loading capacity (LC) of the SLMs are shown in Table 4. The encapsulation efficiencies were in the range of 47.55–81.42 % for SLMs containing 1 %w/w of MN and 83.33–88.05 % for those made with 3 %w/w of MN. So, the SLMs loaded with 3.0 %w/w MN had higher EE%, while those loaded with 1 %w/w MN had the least. However, all batches of the SLMs had good EE% (47.55–88.05 %). Similarly, the loading capacity of the SLMs increased in the same manner. Table 4 shows that maximum LC of 25.99, 16.45, and 25.00 g of MN per 100 g of lipid were obtained for SLMs containing 1, 2, and 3 %w/w of MN, respectively. Thus SLMs containing 3.0 %w/w MN gave higher LC than those containing 1.0 %w/w or 2.0 %w/w MN.

High percentage recoveries from the formulations are a strong indication that the formulation technique adopted was reliable, which is in perfect agreement with earlier studies on SLMs using the melt-emulsification technique [38, 48]. The results of the encapsulation efficiency (EE%) and loading capacity (LC) of the SLMs indicate that generally, EE% and LC increased with increase in drug loading. The ability of the SLMs to accommodate active molecules is a crucial property which can be expressed by the EE% and LC. While EE% defines the ratio between the weight of entrapped API and the total weight of API added to the dispersion, LC expresses the ratio between the entrapped API and the total weight of the lipids [38]. Both EE% and LC are dependent on several parameters, including the lipophilic properties of the API and excipients as well as the formulation method adopted [32]. The EE% and LC were found to be directly proportional to concentration of MN added. Therefore, SLMs with the highest drug concentrations (AM_3_–DM_3_) generally exhibited maximum EE% and LC values. The varied EE% may be as a result of API and matrix physicochemical and material characteristics [38]. Miconazole nitrate is highly lipophilic in nature [23–25]. The lipid contents might have improved the EE% of MN in the SLMs, consistent with previous reports on lipid-based delivery systems of the drug [16, 17, 30]. Moreover, when added in high concentrations, PEG-lipids induce formation of mixed micelles [36]. This could favour drug encapsulation in lipid-based systems such as SLMs.

### 3.5. Continuous Shear Viscometry

Figure 3 shows the viscosity profiles of optimized SLMs. However, MN-loaded SLMs showed a slight increase in viscosity at 12 rpm, followed by a continuous decrease in viscosity at 30 and 60 rpm. It is obvious from Figure 3 that increase in stirring speed (shear rate) led to continuous decrease in viscosity of the SLMs and a continuous increase in the twisting force (torque, which is synonymous with shear stress in rheological parlance). It was observed that MN-loaded SLMs were more viscous than unloaded SLMs and among MN-loaded SLMs, PEGylated SLMs were more viscous than non-PEGylated SLMs. The results equally revealed that batch CM_3_ containing 3 %w/w of MN and 2 %w/w of PEG 4000 had the greatest viscosity values at 6 and 60 rpm but batch BM_3_ containing 3 %w/w of MN and 1 %w/w of PEG 4000 recorded the greatest viscosity values at 12 and 30 rpm.

Results of the viscosity measurement indicate that generally, the SLMs are pseudoplastic or structurally viscous systems; that is to say, they exhibit a strong pseudoplasticity (decrease in viscosity with an increase in shear rate). In other words, the applied force acted on the SLMs and caused the particle sizes to change, the particles to be oriented in the direction of flow or an agglomerate to be dissolved, consistent with a previous rheological study on topical lipid particulate system encapsulating antifungal agent [20]. The enhanced viscosity of the PEGylated SLMs compared with non-PEGylated SLMs may be related to the additional PEG 4000 content in the formulation compositions of the former. Viscosity of vaginal SLMs is an important factor to consider in evaluation of drug deposition in or penetration across the vaginal mucosa and is also used to measure the spreadability, pourability, syringeability, pumpability, flowability, and extrudability of the SLMs. The decreased viscosity of the SLMs with increasing shear rates (and consequently shear stresses) not only implies that these SLM formulations would be easily extruded from their containers or packages but also indicates potential improvement in spreadability for efficient contact with topical infectious organisms such as* C. albicans *(implicated in VVC) as well as enhancement in the diffusivity of the drugs within the polymer-lipid (PEG-lipid) network which in turn would facilitate flux, consistent with previous reports [38, 40].

### 3.6. Morphological Characterization

Figure 4 shows the polarized light micrographs of representative batches of miconazole nitrate-loaded SLMs as well as unloaded SLMs. The results indicate that generally, MN-loaded SLMs were bigger than the API-free SLMs.

Particle micrograph of the formulations showed structured spherical and more discrete smaller but uniformly distributed particle sizes spread all over the formulation. Moreover, absence of particle aggregation in the formulation is indicative of physical stability of the formulations [38, 48]. It should be noted that it is not possible to determine the particle size distribution in a freeze-fractured preparation because the matrix lipid does not recrystallize under the condition, hence, the orientation of the SLMs particles as circular or somewhat cuboidal, whitish structures when viewed edgeon. It also showed that there was no droplet aggregation in the formulations, indicative of physical stability.

### 3.7. FT-IR Spectroscopic Analysis of MN-Loaded SLMs

The FT-IR spectra of MN showed principal characteristic absorption bands of MN described previously. The FT-IR spectrum of miconazole nitrate-loaded SLMs (Figure 2(d)) showed principal characteristic absorption bands of MN at 3809 cm^−1^, 3639 cm^−1^, and 3509 cm^−1^ (N-H stretching), 3334 cm^−1^ and 3188 cm^−1^ (OH group), 2919 cm^−1^, 2728 cm^−1^, and 2616 cm^−1^ (C-H stretching), 2454 cm^−1^ (C=N stretching), 1987 cm^−1^ and 1850 cm^−1^ (carbonyl CHO group), 1605 cm^−1^ (conjugated *α*,*β*-unsaturated C=C bond in benzene ring), 1373 cm^−1^ (NO_2_: nitro group), 1065 cm^−1^ (C-O ester in benzene ring), and 838 cm^−1^ and 745 cm^−1^ (aromatic C-Cl deformation). The results of FT-IR analysis of MN-loaded SLMs also imply that the MN was molecularly dispersed in the SLMs. Overall FT-IR results suggested absence of any incompatibility between MN and excipients used in the formulation [23].

### 3.8. IZDs Determination

Table 4 shows the susceptibility of* C. albicans* to SLMs containing MN. All batches of MN-loaded SLMs produced very significant (p < 0.05) IZDs against the test organism compared with unloaded SLMs, which served as negative controls, consistent with earlier reports [32, 38–41]. SLMs containing 3.0 %w/w of MN (subbatches AM_3_–DM_3_) gave the highest IZDs (25.87 ± 0.94 to 26.33 ± 0.94 mm) against the test organism, while those containing 1.0 %w/w of MN (subbatches AM_1_–DM_1_) gave the least (24.00 ± 0.00 to 24.33 ± 0.42 mm). More so, the test microorganism recorded higher susceptibility to MN-loaded PEGylated SLMs than to MN-loaded non-PEGylated SLMs. Overall, subbatches containing the highest amounts of PEG 4000 and MN gave the greatest IZDs (26.33 ± 0.94 mm for MN-loaded SLMs subbatch DM_3_).

Microbiological test was carried out to determine the sensitivity of the organism (*C. albicans*) to MN-loaded SLMs and to establish that the drug did not lose activity during formulation. MN has established activity against* C. albicans* [24, 49]. It was observed that the greater the amount of MN loaded into the SLMs, the greater the IZD produced, in perfect agreement with previous report [49]. Thus, the formulations exhibited capacity limited bioactivity. Plain SLMs (A_0_–D_0_) did not produce IZDs since they did not contain any drug. High IZDs recorded against* C. albicans* especially with batches AM_3_–DM_3_ were an indication that these formulations may have exhibited the fastest release of the entrapped drug, hence the fast antifungal activity. Presence of surfactants and solubilizers such as Soluplus®, Tween® 80, and PEG 4000 may have resulted to improved solubilization of MN in the lipid core, in line with earlier reports [38, 40]. In furtherance to that, when added in high concentration, PEG-lipids induce formation of mixed micelles [36, 37], which could favour drug encapsulation efficiency in lipid-based systems such as SLMs, and this could ultimately influence the bioactivity of the encapsulated drug [32, 41]. Above all, PEG 4000 being a penetration enhancer might have also facilitated the release of entrapped drugs in PEGylated SLMs from the lipid core and their subsequent transport across the fungal membrane more than the non-PEGylated SLMs. This enhanced anticandidal activity of MN conferred by the SLMs would be of immense benefit in the treatment of candida infections such as VVC caused by MN-susceptible* C. albicans*, consistent with earlier reports on LBDDS containing MN [16, 17, 27, 30, 49].

### 3.9. MIC and MFC Determination

Table 5 shows the MICs and MFCs of optimized MN-loaded SLMs. As could be seen from that table, the antifungal effect of PEGylated MN-loaded SLMs was significantly (p < 0.05) greater than those of non-PEGylated MN-loaded SLMs, Fungusol® topical lotion, and pure MN sample. In addition, while MN-loaded SLMs batches BM_3_ and DM_3_ on one hand and MN-loaded SLMs batch AM_3_ and Fungusol® topical lotion on the other hand exhibited equal antifungal effects, respectively, MN-loaded SLM batch CM_3_ and pure MN sample gave the greatest and least antifungal effects, respectively.

Low MIC and MFC values imply strong antimicrobial properties [44, 45, 47]. The presence of PEG 4000, a hydrophilic surfactant, in the PEGylated SLMs containing MN might have improved the solubilization of the drug in the lipid core. The use of PEG to enhance solubilization has severally been reported [32, 36, 37]. The strong fungicidal efficacy of the SLMs containing MN could also be related to improved solubilization conferred on the drug by the surfactant properties of Soluplus® and Tween® 80 as well as the membrane penetration-enhancing property of PEG 4000 [38, 41, 50].

### 3.10. Time-Kill Assay

**Figure 5:**
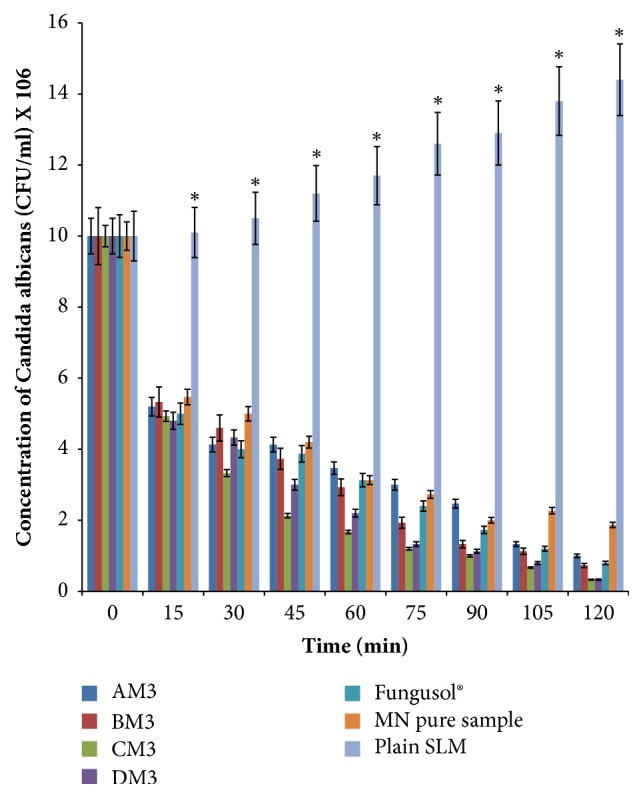
Time-kill assay results for optimized miconazole nitrate-loaded SLMs. Key: AM_3_ is optimized non-PEGylated SLMs containing 3.0 %w/w of miconazole nitrate, BM_3_–DM_3_ are PEGylated SLMs containing increasing concentrations (1.0, 2.0, and 4.0 %w/w) of PEG 4000, respectively, and 3.0 %w/w of miconazole nitrate. Fungusol® is commercial miconazole nitrate-containing topical lotion. Plain SLM means unloaded SLM while MN pure sample is pure miconazole nitrate powder. *∗* Significant at p ≤ 0.05.

Results of time-kill assays of the optimized MN-loaded SLMs against* C. albicans *are presented in Figure 5. At zero hour, the concentration of* C. albicans *for all samples was 10 x 10^6^ cfu/ml. Within 15 min, the fungal viability was in the range of 4.8 x 10^6^–5.47 x 10^6^ cfu/ml. Then killing continued time dependently and within 120 min, the fungal burden decreased to 3.3 x 10^5^–1.87 x 10^6^ cfu/ml. At 120 min, MN-loaded SLMs batches CM_3_ and DM_3_ reduced the fungal viability to 3.3 x 10^5^ cfu/ml. As shown in Table 6 the cell-killing rate parameters (death rate constant and K and D-value) varied with the compositions of the MN-containing samples, with batches CM_3_ and DM_3_, which had 2 and 4 %w/w of PEG 4000, respectively, giving the highest death rate constant and lowest D-value within 120 min of study. Thus, within the 120 min of time-kill assay study, all the MN-containing formulations (including PEGylated and non-PEGylated SLMs as well as Fungusol® lotion ) reduced the fungal viability by 90 % unlike the unformulated drug (pure MN sample) which would require additional time (approximately 45 min) to achieve the same result.

Furthermore, it was observed that the formulations exhibited a fast and powerful fungicidal activity compared with negative control (plain SLMs) that showed consistent increase in fungal burden throughout the study period. Time-dependent killing occurred with the formulations, with approximately 50 % reduction in fungal viability within 15 min (p ≤ 0.05) and nearly complete killing within 120 min. Meanwhile, high death rate constants and low D-values are indicative of strong and powerful microbiocidal activity and* vice versa* [47]. Overall, the time-kill assay revealed that optimized MN-loaded SLMs gave faster and more powerful fungicidal activity than did pure MN sample. However, Fungusol® lotion was more fungicidal than non-PEGylated MN-loaded SLM (batch AM_3_). In addition, time-kill assays also confirmed that MN-loaded PEGylated SLMs had stronger fungicidal activity than their non-PEGylated counterparts. The observed fast-acting and strong fungicidal activity of MN-loaded SLMs could be related to improved lipid solubility conferred on the drugs by the surfactant properties of Soluplus® and Tween® 80, which ensured sustained delivery of the drugs through gradual and sustained erosion of the lipid matrix coupled with the penetration-enhancing property of PEG 4000 [36, 37, 41, 50]. The developed formulation could be useful alternative to Fungusol® topical lotion in the treatment of fungal infections caused by MN-susceptible microorganisms such as* C. albicans *(implicated in VVC).

### 3.11. Stability Studies

**Figure 6:**
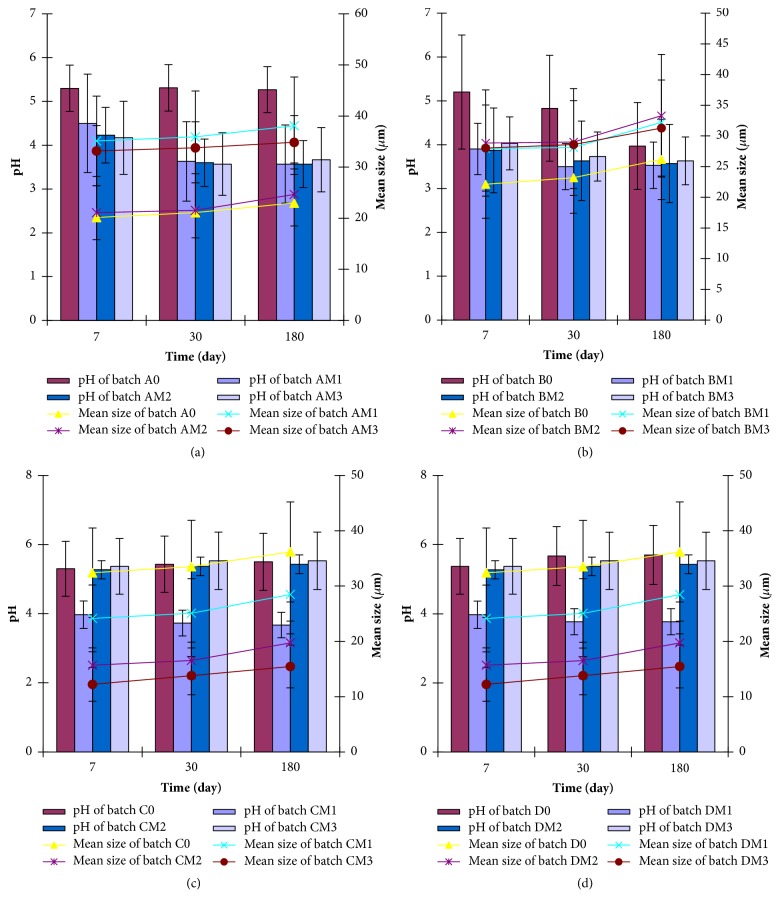
Time-resolved stability of MN-SLMs (A_0_–AM_3_) batches (a), MN-SLMs (B_0_–BM_3_) batches (b), MN-SLMs (C_0_–CM_3_) batches (c), and MN-SLMs (D_0_–DM_3_) batches (d) at room temperature. Key: batches A_0_ and AM_1_–AM_3_ are non-PEGylated SLMs; batches B, C, and D are PEGylated SLMs containing increasing concentrations (1.0, 2.0, and 4.0 %w/w) of PEG 4000, respectively; A_0_–D_0_ are unloaded SLMs while batches AM_1_–DM_1_, AM_2_–DM_2_, and AM_3_–DM_3_ contain increasing concentrations (1.0, 2.0, and 3.0 %w/w) of miconazole nitrate, respectively.

The stability results of the optimized SLMs studied by pH- and size-dependent time-resolved analyses are shown in Figure 6. The particle sizes of both unloaded and MN-loaded SLMs were generally small but increased insignificantly (p > 0.05) with time. It was observed, however, that lipid matrix composition, drug loading, PEGylation, and drug-lipid matrix ratio used in the formulations had insignificant (p > 0.05) effect on the particle size of the SLMs as shown in Figure 6. The results of the time-dependent pH stability studies (Figure 6) showed that, at day 1, the pH of the unloaded SLMs ranged from 5.20 ± 0.10 to 5.37 0.06; the pH of MN-loaded non-PEGylated SLMs ranged from 4.17 ± 0.03 to 4.50 ± 0.10, while the pH values were in the range of 3.87 ± 0.06–4.03 ± 0.06, 3.97 ± 0.10–4.17 ± 0.06, and 3.97 ± 0.06–4.17 ± 0.06 for MN-loaded PEGylated SLMs batches BM_1_–BM_3_, CM_1_–CM_3_, and DM_1_–DM_3_, respectively. With the exception of the pH of unloaded SLMs batch B_0_ that decreased significantly (p < 0.05) with time, the pH of the rest of the formulations remained stable in the acidic region throughout the study. This would discourage VVC infection. However, a slight increase or decrease was noted at 30 and 90 days for some formulations, but this was rather insignificant (p > 0.05).

The stability studies were performed to determine the stability of the different batches of the SLMs when stored at room temperature and at different time intervals. The particle sizes of the SLMs are within the acceptable range for dermal/topical applications [20, 28, 29, 49]. Particle size may be a function of formulation excipients, degree of homogenization, homogenization pressure, rate of particle size growth, crystalline habit of the particle, etc. [32]. The increase of the particles over time may be due to the additive interacting forces [electrical repulsion forces (V_R_) and the van der Waals attraction (V_A_)] existing between the particles [51]. The interparticulate distance in the SLMs is appreciably small thereby allowing the predomination of the van der Waals universal attractive forces giving rise to larger particles on aggregation over time. This agrees with earlier studies on SLMs [38, 48]. Furthermore, increase in particle size could be associated with aggregation and subsequent growth by Ostwald ripening or sintering [32]. Because of the apparent particle size stability obtained after 180 days, these systems could be adjudged to be relatively stable. Additionally, microscopic studies show that many disperse systems, e.g., emulsions, flocculate a few second after preparation [32]. Also, many particle size analyses require a diluted sample, and this dilution in many cases causes flocs to break-up. In this study, dilution of sample was not done and the particle sizes obtained reflect the true particle sizes of the particles at the time of analysis. Homolipids in combination with mobile surfactants have been shown to produce very stable disperse systems [41, 48, 52].

## 4. Conclusion

The use of MN for localized treatment of VVC is limited by low bioavailability, requirement for multiple daily doses, difficulty in reaching the target site due to the low penetration capacity to treat deep-seated recurrent VVC, and drug resistance. In this study, the potential of intravaginal PEGylated SLMs in prolonged localized vaginal delivery of MN was investigated for improved treatment of VVC. To this end, vaginal tailor-made SLMs encapsulating MN were developed using structured phytolipids (SF and SO) and PEG 4000 and evaluated for improved localized treatment of VVC. Solid state characterization performed on the lipid matrices used in preparing the formulations confirmed the suitability of the lipid matrices and their compatibility with MN. SLMs with physicochemical properties suitable for vaginal drug delivery were obtained. PEGylated SLMs gave better physicochemical properties than non-PEGylated SLMs and formulations made with 4 %w/w of the polymer (PEG 4000) and containing 3 %w/w MN were pseudoplastic and safe upon vaginal application and demonstrated improved* in vitro* performance compared with unformulated drug sample (MN pure sample) and commercial topical formulation of MN (Fungusol® lotion). This study has shown that intravaginal PEGylated SLMs represent a promising approach for improving the delivery and localized antifungal activity of MN for effective treatment of VVC, thus necessitating further development of the formulation as mucoadhesive carrier system.

## Figures and Tables

**Table 1:** Formulation composition of lipid matrices for SLMs preparation.

Sample	Ingredients (%w/w)
LM 1:1	Super-refined sunseed oil (50) and Softisan® 154 (50)
LM 1:3	Super-refined sunseed oil (33.33) and Softisan® 154 (66.67)
LM 1:6	Super-refined sunseed oil (16.67) and Softisan® 154 (83.33)
LM 1:9	Super-refined sunseed oil (10) and Softisan® 154 (90)
PEG-LM 1:1 (1:9)	PEG 4000 (10) and Lipid matrix, LM 1:1 (90)
PEG-LM 1:1 (2:8)	PEG 4000 (20) and Lipid matrix, LM 1:1 (80)
PEG-LM 1:1 (4:6)	PEG 4000 (40) and Lipid matrix, LM 1:1 (60)
PEG-LM 1:3 (1:9)	PEG 4000 (10) and Lipid matrix, LM 1:3 (90)
PEG-LM 1:3 (2:8)	PEG 4000 (20) and Lipid matrix, LM 1:3 (80)
PEG-LM 1:3 (4:6)	PEG 4000 (40) and Lipid matrix, LM 1:3 (60)
PEG-LM 1:6 (1:9)	PEG 4000 (10) and Lipid matrix, LM 1:6 (90)
PEG-LM 1:6 (2:8)	PEG 4000 (20) and Lipid matrix, LM 1:6 (80)
PEG-LM 1:6 (4:6)	PEG 4000 (40) and Lipid matrix, LM 1:6 (60)
PEG-LM 1:9 (1:9)	PEG 4000 (10) and Lipid matrix, LM 1:9 (90)
PEG-LM 1:9 (2:8)	PEG 4000 (20) and Lipid matrix, LM 1:9 (80)
PEG-LM 1:9 (4:6)	PEG 4000 (40) and Lipid matrix, LM 1:9 (60)

Key: LM means lipid matrix prepared with super-refined sunseed oil and Softisan® 154; LM 1:1, LM 1:3, LM 1:6, and LM 1:9 are non-PEGylated lipid matrices containing increasing amounts of Softisan® 154; PEG-LM 1:1(1:9), PEG-LM 1:1(2:8), and PEG-LM 1:1(4:6) are PEGylated lipid matrices based on 1:1 ratio of sunseed oil and Softisan® 154 and containing increasing amounts of PEG 4000; PEG-LM 1:3(1:9), PEG-LM 1:3(2:8), and PEG-LM 1:3(4:6) are PEGylated lipid matrices based on 1:3 ratio of sunseed oil and Softisan® 154 and containing increasing amounts of PEG 4000; PEG-LM 1:6(1:9), PEG-LM 1:6(2:8), and PEG-LM 1:6(4:6) are PEGylated lipid matrices based on 1:6 ratio of sunseed oil and Softisan® 154 and containing increasing amounts of PEG 4000, while PEG-LM 1:9(1:9), PEG-LM 1:9(2:8), and PEG-LM 1:9(4:6) are PEGylated lipid matrices based on 1:9 ratio of sunseed oil and Softisan® 154 and containing increasing amounts of PEG 4000.

**Table 2:** Formulation composition of miconazole nitrate-loaded SLMs.

Batch code	Lipid matrix (%w/w)	Miconazole nitrate (%w/w)	Tween® 80 (%w/w)	Soluplus® (%w/w)	Sorbic acid (%w/w)	Distilled water q.s. to 100 %w/w
AM_1_	10.0	1.0	1.0	4.0	0.1	100.0
BM_1_	10.0	1.0	1.0	4.0	0.1	100.0
CM_1_	10.0	1.0	1.0	4.0	0.1	100.0
DM_1_	10.0	1.0	1.0	4.0	0.1	100.0
AM_2_	10.0	2.0	1.0	4.0	0.1	100.0
BM_2_	10.0	2.0	1.0	4.0	0.1	100.0
CM_2_	10.0	2.0	1.0	4.0	0.1	100.0
DM_2_	10.0	2.0	1.0	4.0	0.1	100.0
AM_3_	10.0	3.0	1.0	4.0	0.1	100.0
BM_3_	10.0	3.0	1.0	4.0	0.1	100.0
CM_3_	10.0	3.0	1.0	4.0	0.1	100.0
DM_3_	10.0	3.0	1.0	4.0	0.1	100.0
A_0_	10.0	0.0	1.0	4.0	0.1	100.0
B_0_	10.0	0.0	1.0	4.0	0.1	100.0
C_0_	10.0	0.0	1.0	4.0	0.1	100.0
D_0_	10.0	0.0	1.0	4.0	0.1	100.0

Key: batches (A_0_, AM_1_–AM_3_) are non-PEGylated SLMs; batches B, C, and D are PEGylated SLMs containing increasing concentrations (1.0, 2.0, and 4.0 %w/w) of PEG 4000, respectively; A_0_–D_0_ are unloaded SLMs while batches AM_1_–DM_1_, AM_2_–DM_2_, and AM_3_–DM_3_ contain increasing concentrations (1.0, 2.0, and 3.0 %w/w) of miconazole nitrate, respectively.

**Table 3:** Thermal properties of the lipid matrices for SLMs preparation.

Sample	Melting point (°C)	Enthalpy (W/g)	Melting temperature range (°C)	Area (J/g)
Softisan® 154	61.40	- 8.9	37 – 75	71.92
LM 1:1	60.82	- 9.0	25 – 75	271.5
LM 1:3	61.62	- 2.5	25 – 75	61.19
LM 1:6	115.18	- 0.25	110 – 127	4.228
LM 1:9	64.56	- 2.05	25 – 75	47.63
PEG 4000	64.40	- 3.6	50 – 75	67.20
PEG-LM1:1 (1:9)	142.49	- 0.35	135 – 157	33.24
PEG-LM1:1 (2:8)	145.66	- 0.4	142 – 148	47.18
PEG-LM1:1 (4:6)	62.66	- 2.0	25 – 75	64.87
PEG-LM1:3 (1:9)	88.00	- 0.35	80 – 115	25.35
PEG-LM1:3 (2:8)	60.60	- 3.4	45 – 70	39.89
PEG-LM1:3 (4:6)	65.76	- 2.3	25 – 75	53.63
PEG-LM1:6 (1:9)	61.51	- 4.0	37 – 75	84.55
PEG-LM1:6 (2:8)	61.57	- 3.25	25 – 75	52.22
PEG-LM1:6 (4:6)	61.75	- 4.5	25 – 75	99.11
PEG-LM1:9 (1:9)	60.75	- 4.25	25 – 75	72.59
PEG-LM1:9 (2:8)	61.32	- 3.6	25 – 75	60.29
PEG-LM1:9 (4:6)	61.12	- 4.1	25 – 75	68.82

Key: LM 1:1, LM 1:3, LM 1:6, and LM 1:9 are non-PEGylated lipid matrices containing increasing amounts of Softisan® 154; PEG-LM 1:1(1:9), PEG-LM 1:1(2:8), and PEG-LM 1:1(4:6) are PEGylated lipid matrices based on 1:1 ratio of sunseed oil and Softisan® 154 and containing increasing amounts (10, 20, and 40 %w/w) of PEG 4000; PEG-LM 1:3(1:9), PEG-LM 1:3(2:8), and PEG-LM 1:3(4:6) are PEGylated lipid matrices based on 1:3 ratio of sunseed oil and Softisan® 154 and containing increasing amounts (10, 20, and 40 %w/w) of PEG 4000; PEG-LM 1:6(1:9), PEG-LM 1:6(2:8), and PEG-LM 1:6(4:6) are PEGylated lipid matrices based on 1:6 ratio of sunseed oil and Softisan® 154 and containing increasing amounts (10, 20, and 40 %w/w) of PEG 4000, while PEG-LM 1:9(1:9), PEG-LM 1:9(2:8), and PEG-LM 1:9(4:6) are PEGylated lipid matrices based on 1:9 ratio of sunseed oil and Softisan® 154 and containing increasing amounts (10, 20, and 40 %w/w) of PEG 4000.

**Table 4:** Some physicochemical and anticandidal properties of MN-loaded SLMs.

Batch code	Yield (%)	Encapsulation efficiency (%)	Loading capacity (g API/100 g lipid)	Inhibition zone diameter (mm±SD)
A_0_	97.90	-	-	-
AM_1_	97.58	54.01	5.40	24.00 ± 0.00
AM_2_	98.22	77.38	15.47	24.67 ± 0.47
AM_3_	98.40	87.75	26.33	25.87 ± 0.94
B_0_	98.92	-	-	-
BM_1_	99.02	73.61	25.99	24.00 ± 0.00
BM_2_	96.54	81.42	16.28	25.00 ± 0.00
BM_3_	97.90	86.64	7.36	26.00 ± 0.00
C_0_	97.44	-	-	-
CM_1_	94.76	81.42	8.14	24.33 ± 0.47
CM_2_	98.10	82.26	16.45	25.67 ± 0.47
CM_3_	99.52	83.05	24.92	26.33 ± 0.47
D_0_	98.26	-	-	-
DM_1_	96.52	47.55	4.76	24.33 ± 0.47
DM_2_	97.80	64.11	12.82	25.83 ± 0.94
DM_3_	97.66	83.33	25.00	26.33 ± 0.94

Keys: A_0_, AM_1_, AM_2_, and AM_3_ are non-PEGylated SLMs; batches B–D are PEGylated SLMs containing increasing concentrations (1.0, 2.0, and 4.0 %w/w) of PEG 4000, respectively; A_0_–D_0_ are unloaded SLMs while batches AM_1_–DM_1_, AM_2_–DM_2_, and AM_3_–DM_3_ contain increasing concentrations (1.0, 2.0, and 3.0 %w/w) of miconazole nitrate (MN), respectively.

**Table 5:** MICs and MFCs of optimized miconazole nitrate-loaded SLMs.

Formulation code	MIC (*μ*g/ml)	MFC (*μ*g/ml)
AM_3_	29.297 ± 2.65	468.754 ± 5.06
BM_3_	14.648 ± 0.98	234.375 ± 4.33
CM_3_	7.324 ± 1.32	117.188 ± 2.19
DM_3_	14.648 ± 3.08	234.375 ± 4.25
MN pure sample suspension	58.594 ± 4.11	937.500 ± 3.11
Fungusol® lotion	29.297 ± 2.22	468.754 ± 6.00

Key: MIC and MFC are minimum inhibitory concentration and minimum fungicidal concentration; AM_3_ is optimized non-PEGylated SLMs containing 3.0 %w/w of miconazole nitrate; BM_3_–DM_3_ are PEGylated SLMs containing increasing concentrations (1.0, 2.0, and 4.0 %w/w) of PEG 4000, respectively, and 3.0 %w/w of miconazole nitrate; Fungusol® is commercial topical formulation of miconazole nitrate, while MN is pure miconazole nitrate sample.

**Table 6:** Cell killing parameters of optimized miconazole nitrate-loaded SLMs.

Formulation code	Death rate constant, K (min^−1^)	D-value (min)
AM_3_	7.10 x 10^−3^	120.000
BM_3_	8.80 x 10^−3^	105.541
CM_3_	1.09 x 10^−2^	81.026
DM_3_	1.09 x 10^−2^	81.026
Fungusol® lotion	8.00 x 10^−3^	109.389
MN pure sample suspension	5.60 x 10^−3^	164.835

Key: AM_3_ is optimized non-PEGylated SLMs containing 3.0 %w/w of miconazole nitrate; BM_3_–DM_3_ are PEGylated SLMs containing increasing concentrations (1.0, 2.0, and 4.0 %w/w) of PEG 4000, respectively, and 3.0 %w/w of miconazole nitrate; Fungusol® is commercial topical formulation of miconazole nitrate, while MN is pure miconazole nitrate sample.

## Abbreviations

SLM: Solid lipid microparticles
PEG 4000: Polyethylene glycol 4000
SO: Sunseed oil
SF: Softisan® 154
MN: Miconazole nitrate
VVC: Vulvovaginal candidiasis
BCS: Biopharmaceutical classification system
LM: Lipid matrix
PEG-LM: PEGylated lipid matrix
PLM: Polarized light microscopy
LBDDS: Lipid-based drug delivery system
API: Active pharmaceutical ingredient
cfu: Colony forming unit
IZD: Inhibition zone diameter
MIC: Minimum inhibitory concentration
MFC: Minimum fungicidal concentration.

## Supplementary Materials

Supplementary 1
** Supplementary Figure** **1:** DSC thermograms of Softisan® 154 (a), PEG 4000 (b), and non-PEGylated lipid matrices based on super-refined sunseed oil and Softisan® 154 in ratios 1:1 (c), 1:3 (d), 1:6 (e), and 1:9 (f).

Supplementary 2
** Supplementary Figure** **2:** DSC thermograms of PEGylated lipid matrices based on 1:1 ratio of sunseed oil and Softisan® 154 and containing increasing amounts (10, 20, and 40 %w/w) of PEG 4000 (a-c), PEGylated lipid matrices based on 1:3 ratio of sunseed oil and Softisan® 154 and containing increasing amounts (10, 20, and 40 %w/w) of PEG 4000 (d-f), PEGylated lipid matrices based on 1:6 ratio of sunseed oil and Softisan® 154 and containing increasing amounts (10, 20, and 40 %w/w) of PEG 4000 (g-i), and PEGylated lipid matrices based on 1:9 ratio of sunseed oil and Softisan® 154 and containing increasing amounts of PEG 4000 (j-l).

Supplementary 3
** Supplementary Figure** **3:** polarized light micrographs (PLM) of miconazole nitrate- (MN-) loaded non-PEGylated and PEGylated lipid matrices showing effective drug solubilization at 3 %w/w (a and b) and static drug crystallization at 5 %w/w (c and d). Note: non-PEGylated lipid matrix (LM 1:9) prepared with super-refined sunseed oil (10 %w/w) and Softisan® 154 (90 %w/w) and PEGylated lipid matrix [PEG-LM 1:9 (4:6)] made with PEG 4000 (40 %w/w) and LM 1:9 (60 %w/w) were used.

Supplementary 4
** Supplementary Figure** **4:** polarized light micrographs (PLM) of SLMs formulated at 5,000 rpm for 10 min (a), 10,000 rpm for 5 min (b), and 10,000 rpm for 7.5 min (c).
