# Supplementary figures and images for: Novel Intravaginal Drug Delivery System Based on Molecularly PEGylated Lipid Matrices for Improved Antifungal Activity of Miconazole Nitrate
Source: Biomed Res Int. 2018 Jun 6;2018:3714329. doi: 10.1155/2018/3714329 (PMC6011106; doi:10.1155/2018/3714329)

Figure 1

| 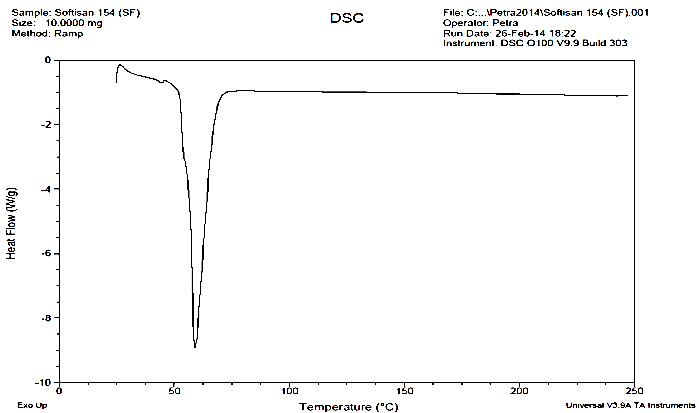 (a) | 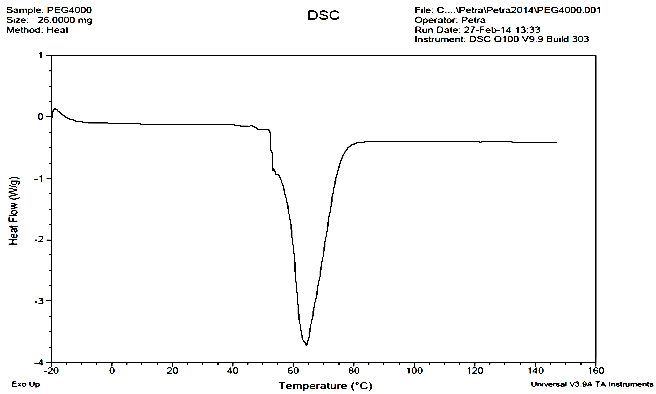 (b) |
| --- | --- |
| 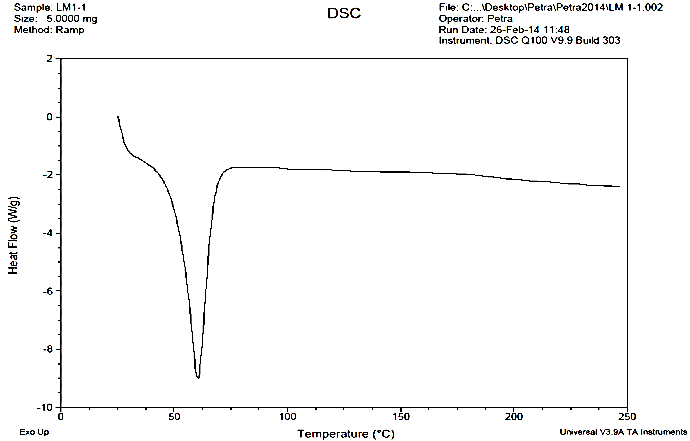 (c) | 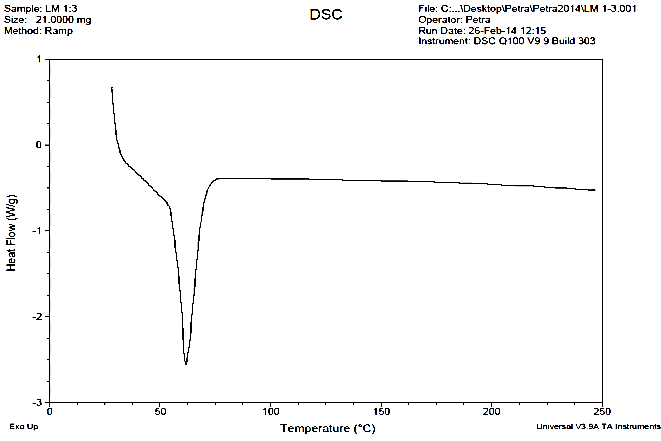 (d) |
| 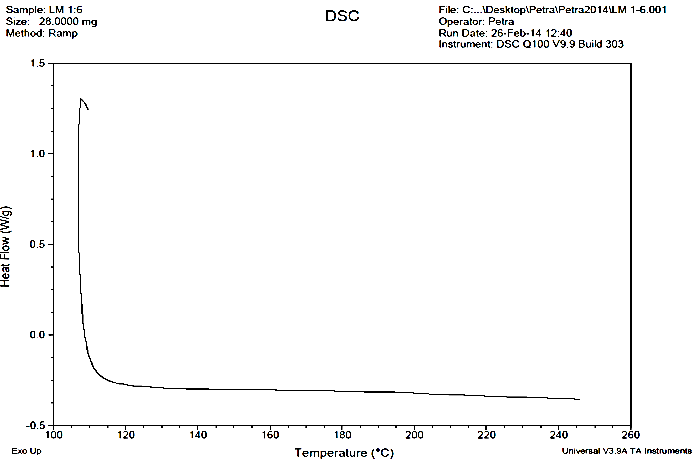 (e) | 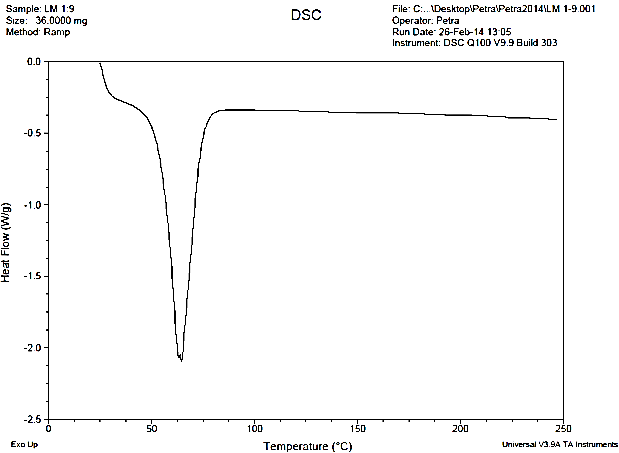 (f) |

Supplement: Supplementary 1 — Supplementary Figure 1: DSC thermograms of Softisan® 154 (a), PEG 4000 (b), and non-PEGylated lipid matrices based on super-refined sunseed oil and Softisan® 154 in ratios 1:1 (c), 1:3 (d), 1:6 (e), and 1:9 (f). [file 3714329.f1.docx]

Figure 2

| 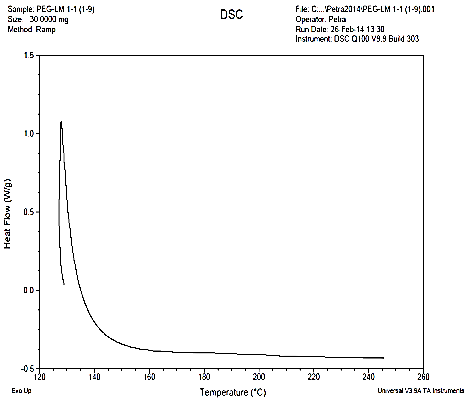 (a) | 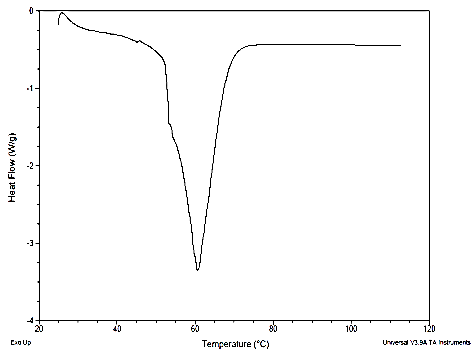 (b) | 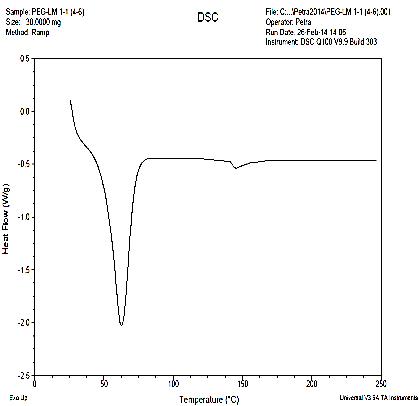 (c) |
| --- | --- | --- |
| 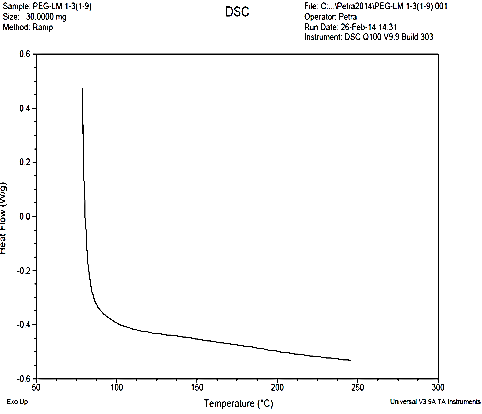 (d) | 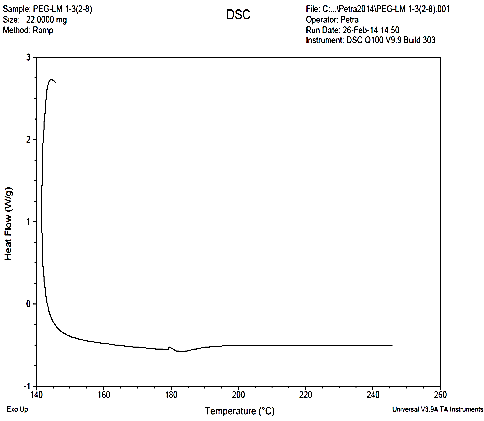 (e) | 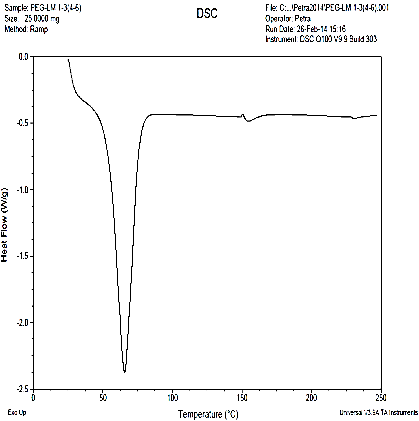 (f) |
| 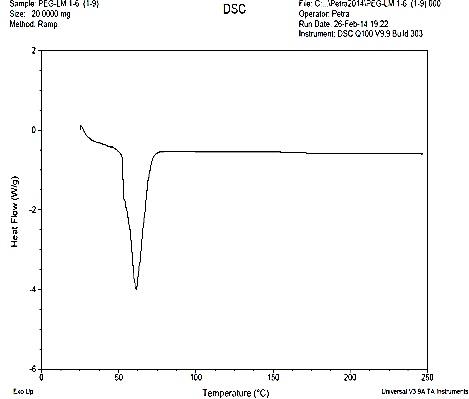 (g) | 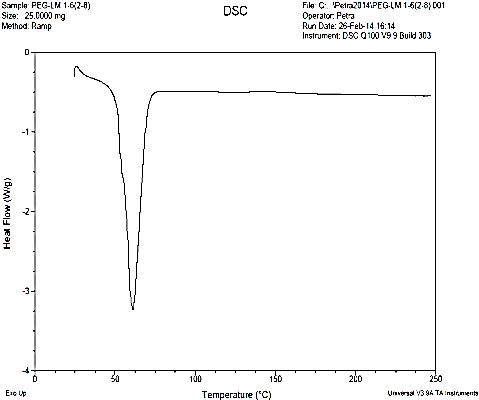 (h) | 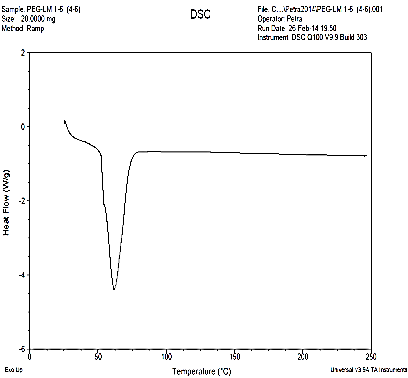 (i) |
| 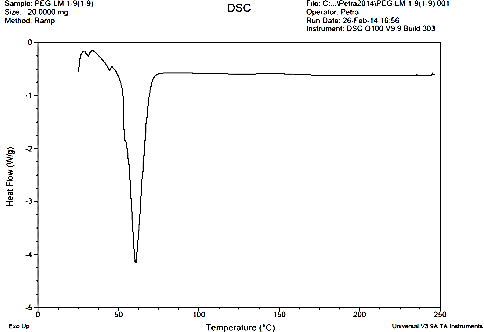 (j) | 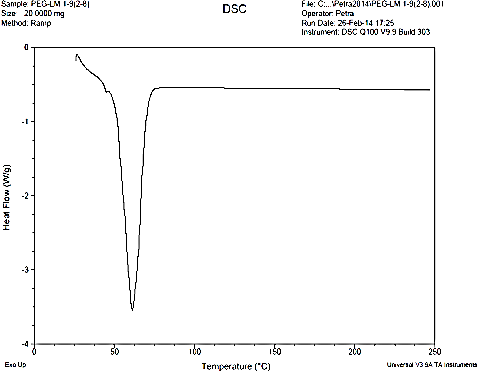 (k) | 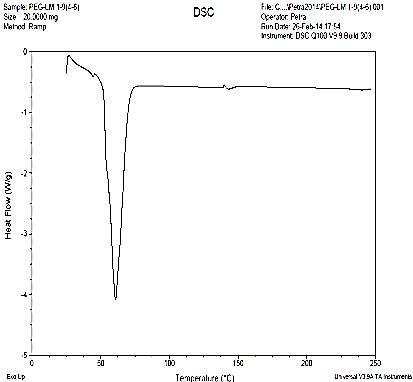 (l) |

Supplement: Supplementary 2 — Supplementary Figure 2: DSC thermograms of PEGylated lipid matrices based on 1:1 ratio of sunseed oil and Softisan® 154 and containing increasing amounts (10, 20, and 40 %w/w) of PEG 4000 (a-c), PEGylated lipid matrices based on 1:3 ratio of sunseed oil and Softisan® 154 and containing increasing amounts (10, 20, and 40 %w/w) of PEG 4000 (d-f), PEGylated lipid matrices based on 1:6 ratio of sunseed oil and Softisan® 154 and containing increasing amounts (10, 20, and 40 %w/w) of PEG 4000 (g-i), and PEGylated lipid matrices based on 1:9 ratio of sunseed oil and Softisan® 154 and containing increasing amounts of PEG 4000 (j-l). [file 3714329.f2.docx]

Figure 3

| (a)  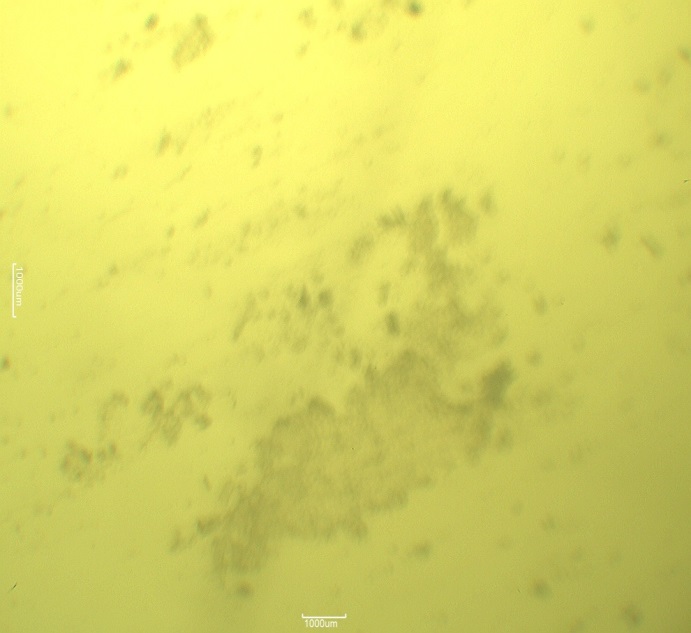 | (b)  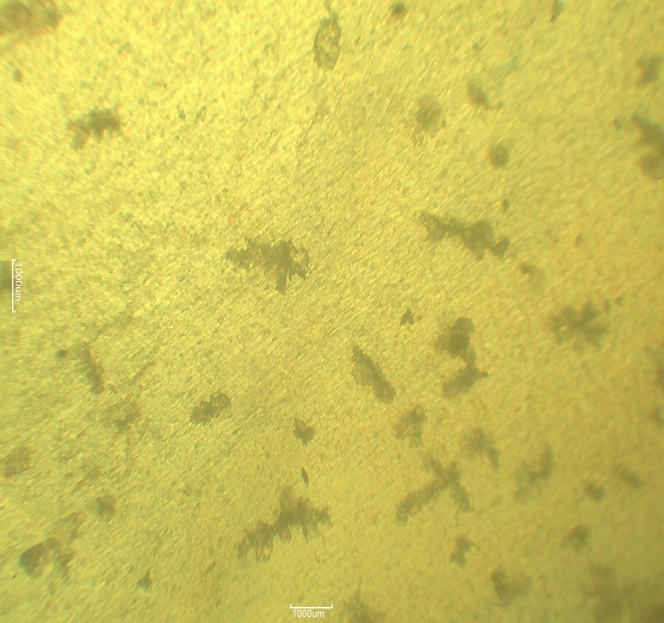 |
| --- | --- |
| (c)  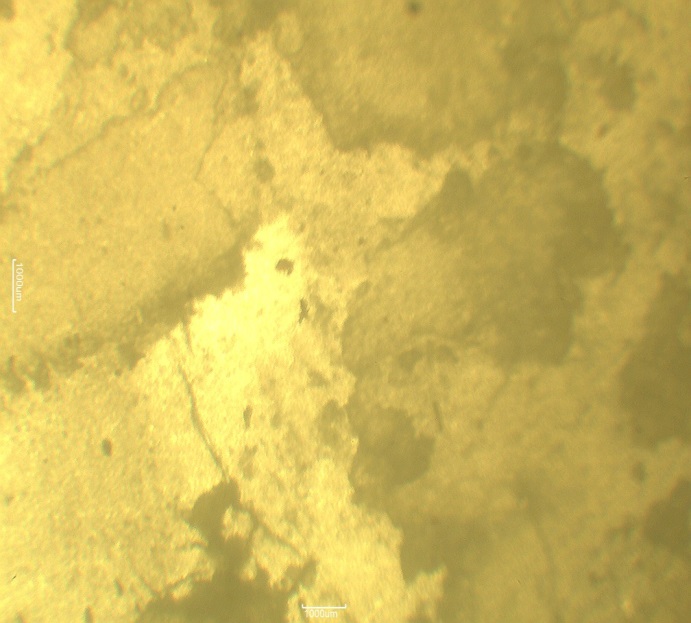 | (d)  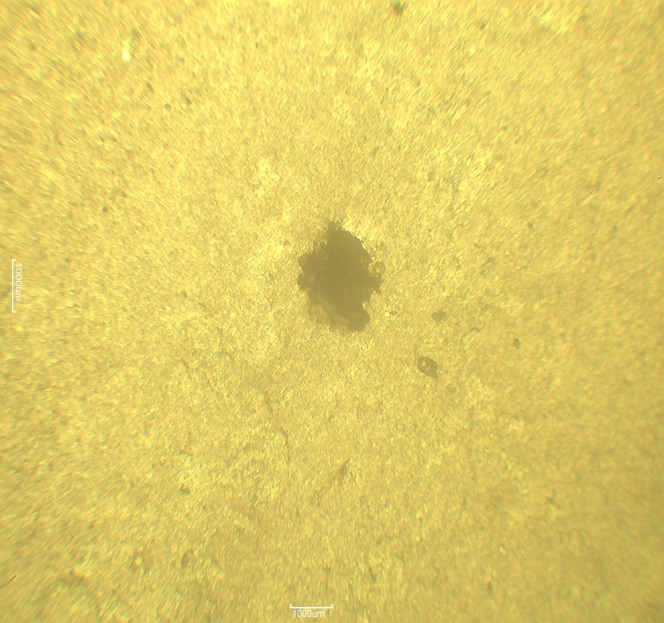 |

Supplement: Supplementary 3 — Supplementary Figure 3: polarized light micrographs (PLM) of miconazole nitrate- (MN-) loaded non-PEGylated and PEGylated lipid matrices showing effective drug solubilization at 3 %w/w (a and b) and static drug crystallization at 5 %w/w (c and d). Note: non-PEGylated lipid matrix (LM 1:9) prepared with super-refined sunseed oil (10 %w/w) and Softisan® 154 (90 %w/w) and PEGylated lipid matrix [PEG-LM 1:9 (4:6)] made with PEG 4000 (40 %w/w) and LM 1:9 (60 %w/w) were used. [file 3714329.f3.docx]

Figure 4

| 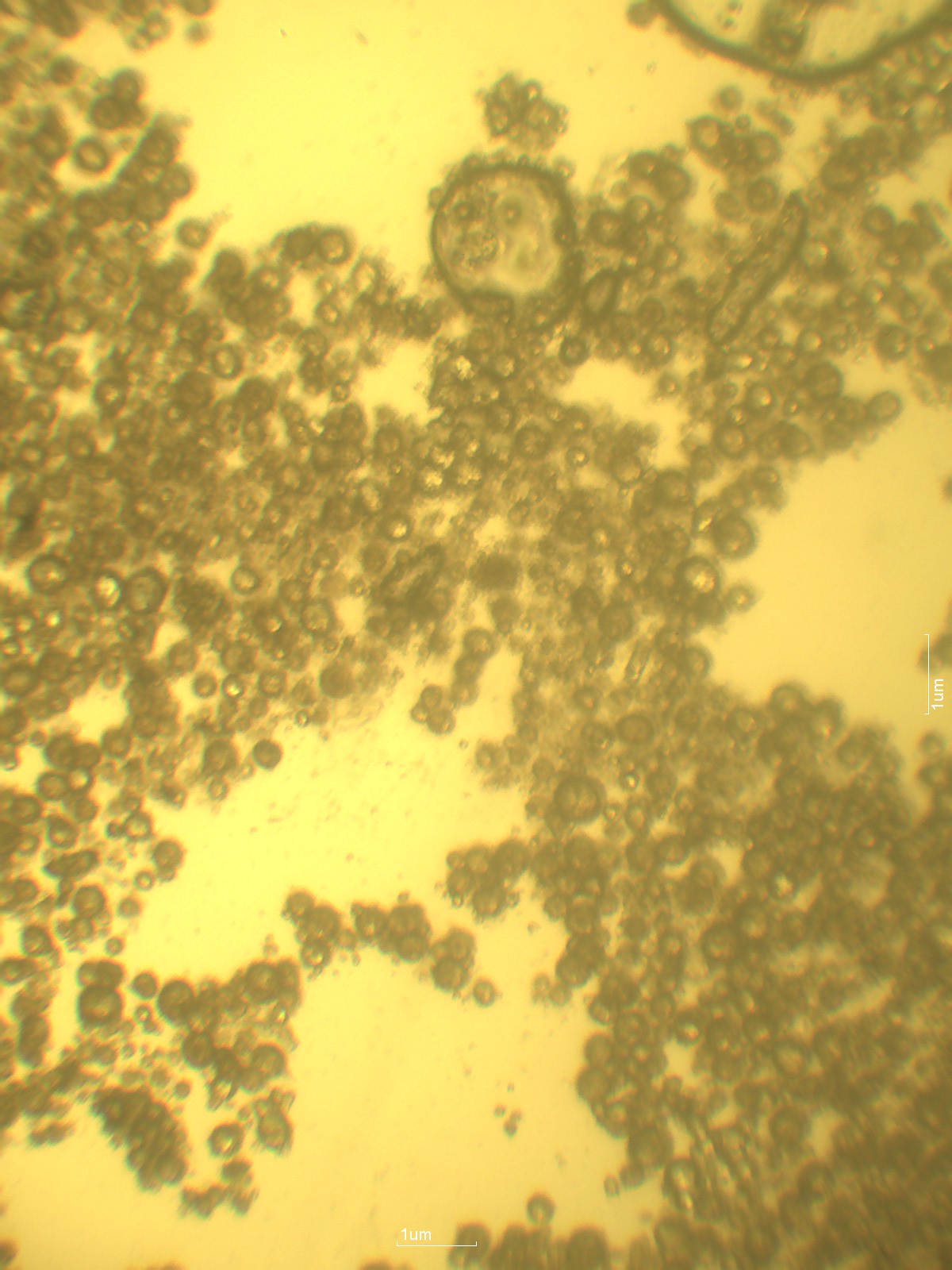  (a) |
| --- |
| 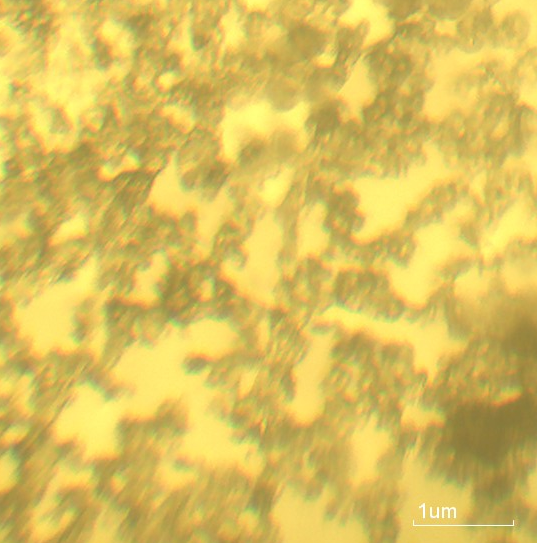  (b) |
| 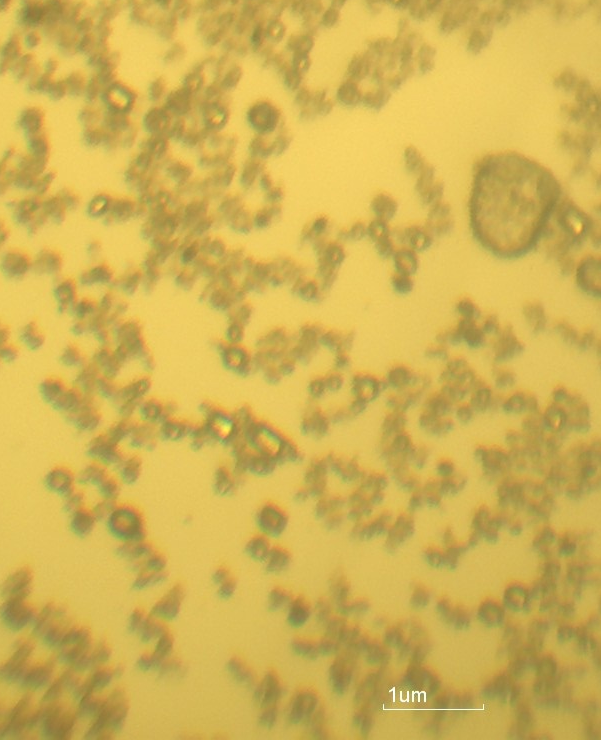  (c) |

Supplement: Supplementary 4 — Supplementary Figure 4: polarized light micrographs (PLM) of SLMs formulated at 5,000 rpm for 10 min (a), 10,000 rpm for 5 min (b), and 10,000 rpm for 7.5 min (c). [file 3714329.f4.docx]
